# A global database of Holocene paleotemperature records

**DOI:** 10.1038/s41597-020-0445-3

**Published:** 2020-04-14

**Authors:** Darrell Kaufman, Nicholas McKay, Cody Routson, Michael Erb, Basil Davis, Oliver Heiri, Samuel Jaccard, Jessica Tierney, Christoph Dätwyler, Yarrow Axford, Thomas Brussel, Olivier Cartapanis, Brian Chase, Andria Dawson, Anne de Vernal, Stefan Engels, Lukas Jonkers, Jeremiah Marsicek, Paola Moffa-Sánchez, Carrie Morrill, Anais Orsi, Kira Rehfeld, Krystyna Saunders, Philipp S. Sommer, Elizabeth Thomas, Marcela Tonello, Mónika Tóth, Richard Vachula, Andrei Andreev, Sebastien Bertrand, Boris Biskaborn, Manuel Bringué, Stephen Brooks, Magaly Caniupán, Manuel Chevalier, Les Cwynar, Julien Emile-Geay, John Fegyveresi, Angelica Feurdean, Walter Finsinger, Marie-Claude Fortin, Louise Foster, Mathew Fox, Konrad Gajewski, Martin Grosjean, Sonja Hausmann, Markus Heinrichs, Naomi Holmes, Boris Ilyashuk, Elena Ilyashuk, Steve Juggins, Deborah Khider, Karin Koinig, Peter Langdon, Isabelle Larocque-Tobler, Jianyong Li, André Lotter, Tomi Luoto, Anson Mackay, Eniko Magyari, Steven Malevich, Bryan Mark, Julieta Massaferro, Vincent Montade, Larisa Nazarova, Elena Novenko, Petr Pařil, Emma Pearson, Matthew Peros, Reinhard Pienitz, Mateusz Płóciennik, David Porinchu, Aaron Potito, Andrew Rees, Scott Reinemann, Stephen Roberts, Nicolas Rolland, Sakari Salonen, Angela Self, Heikki Seppä, Shyhrete Shala, Jeannine-Marie St-Jacques, Barbara Stenni, Liudmila Syrykh, Pol Tarrats, Karen Taylor, Valerie van den Bos, Gaute Velle, Eugene Wahl, Ian Walker, Janet Wilmshurst, Enlou Zhang, Snezhana Zhilich

**Affiliations:** 1 Northern Arizona University, School of Earth and Sustainability, Flagstaff, AZ 86011 USA; 2 University of Lausanne, Institute of Earth Surface Dynamics, Lausanne, 1015 Switzerland; 3 University of Basel, Department of Environmental Sciences, Basel, 4056 Switzerland; 4 University of Bern, Institute of Geological Sciences and Oeschger Center for Climate Change Research, Bern, CH-3012 Switzerland; 5 University of Arizona, Department of Geosciences, Tucson, AZ 85721 USA; 6 University of Bern, Institute of Geography and Oeschger Centre for Climate Change Research, Bern, 3012 Switzerland; 7 Northwestern University, Department of Earth and Planetary Sciences, Evanston, IL 60208 USA; 8 University of Utah, Department of Geography, Salt Lake City, UT 84112 USA; 9 Université de Montpellier, Centre National de la Recherche Scientifique, Institut des Sciences de l’Evolution, Montpellier, 34095 France; 10 Mount Royal University, Department of General Education, Calgary, T3E6K6 Canada; 11 Université du Québec à Montréal, Geotop-UQAM, Montréal, H3C 3P8 Canada; 12 University of London, Birkbeck, Department of Geography, London, WC1E 7HX UK; 13 University of Bremen, MARUM Center for Marine Environmental Sciences, Bremen, 28359 Germany; 14 University of Wisconsin-Madison, Department of Geoscience, Madison, WI 53706 USA; 15 Durham University, Department of Geography, Durham, DH1 3LE UK; 16 University of Colorado, Cooperative Institute for Research in Environmental Sciences, Boulder, CO 80309 USA; 17 Laboratoire des Sciences du Climat et de l’Environnement, Université Paris-Saclay, Gif sur Yvette, 91191 France; 18 Heidelberg University, Institute of Environmental Physics, Heidelberg, 69221 Germany; 19 Australian Nuclear Science and Technology Organisation, Environment, Lucas Heights, 2234 Australia; 20 Institute for Coastal Research, Helmholtz-Zentrum, Geesthacht, Germany; 21 University at Buffalo, Department of Geology, Buffalo, NY 14206 USA; 22 Universidad Nacional de Mar del Plata, Instituto de Investigaciones Marinas y Costeras, Mar del Plata, 7600 Argentina; 23 Balaton Limnological Institute, Centre for Ecological Research, Tihany, H-8237 Hungary; 24 Brown University, Department of Earth, Environmental and Planetary Sciences, Providence, 2912 USA; 25 Alfred Wegener Institut Helmholtz Centre for Polar and Marine Research, Polar Terrestrial Environmental Systems, Potsdam, 14473 Germany; 26 Ghent University, Renard Centre of Marine Geology, Gent, 9000 Belgium; 27 Natural Resources Canada, Geological Survey of Canada, Calgary, AB T2L 2A7 Canada; 28 Natural History Museum, Department of Life Sciences, London, SW7 5BD UK; 29 University of Concepcion, Department of Oceanography and COPAS Sur-Austral Program, Concepcion, 4030000 Chile; 30 University of New Brunswick, Department of Biology, Fredericton, NB E3B 5A3 Canada; 31 University of Southern California, Department of Earth Sciences, Los Angeles, CA 90089 USA; 32 Goethe University, Department of Physical Geography, Frankfurt am Main, 60438 Germany; 33 University of Ottawa, Ottawa-Carleton Institute of Biology, Ottawa, K1N6N5 Canada; 34 Newcastle University, School of Geography, Politics and Sociology, Newcastle-upon-Tyne, NE17RU UK; 35 British Antarctic Survey, Palaeoenvironments and Ice Sheets, Cambridge, CB3 0ET UK; 36 University of Arizona, School of Anthropology, Tucson, AZ 85721 USA; 37 University of Ottawa, Department of Geography, Environment and Geomatics, Ottawa, K1N6N5 Canada; 38 Aquatica GmbH, Bern, 3007 Switzerland; 39 Okanagan College, Department of Geography and Earth and Environmental Science, Kelowna, V1Y 4X8 Canada; 40 Sheffield Hallam University, Department of the Natural and Built Environment, Sheffield, S1 1WB UK; 41 University of Innsbruck, Department of Ecology, Innsbruck, 6020 Austria; 42 University of Southern California, Information Sciences Institute, Marina Del Rey, CA 90292 USA; 43 University of Southampton, School of Geography and Environmental Science, Southampton, SO17 1BJ UK; 44 The LAKES Institute, Lyss, 3250 Switzerland; 45 Northwest University, China, College of Urban and Environmental Sciences, Xi’an, 710027 China; 46 University of Bern, Palaeoecology, Bern, CH-3013 Switzerland; 47 University of Helsinki, Faculty of Biological and Environmental Sciences, Lahti, 15140 Finland; 48 University College London, Department of Geography, London, WC1E 6BT UK; 49 Eötvös Loránd University, Department of Environmental and Landscape Geography, Budapest, 1117 Hungary; 50 The Ohio State University, Department of Geography and Byrd Polar and Climate Research Center, Columbus, OH 43210 USA; 51 CONICET Argentina, CENAC/APN, Bariloche, RN 8400 Argentina; 52 Potsdam University, Institute of Geosciences, Potsdam, 14476 Germany; 53 Lomonosov Moscow State University, Faculty of Geography, Moscow, 119991 Russia; 54 Masaryk University, Department of Botany and Zoology, Brno, 61137 Czech Republic; 55 Bishop’s University, Department of Environment and Geography, Sherbrooke, Quebec J1M 1Z7 Canada; 56 Université Laval, Department of Geography, Center for Northern Studies, Québec, G1V 0A6 Canada; 57 University of Lodz, Department of Invertebrate Zoology and Hydrobiology, Lodz, 90-237 Poland; 58 University of Georgia, Department of Geography, Athens, GA 30606 USA; 59 National University of Ireland Galway, School of Geography, Archaeology and Irish Studies, Galway, H91 TK33 Ireland; 60 Victoria University of Wellington, School of Geography, Environment and Earth Sciences, Wellington, 6012 New Zealand; 61 Sinclair Community College, Geography Department, Dayton, OH 45402 USA; 62 Fisheries and Ocean Canada, Gulf Fisheries Centre, Moncton, NB E1C 9B6 Canada; 63 University of Helsinki, Department of Geosciences and Geography, Helsinki, 00014 Finland; 64 The Natural History Museum, London, SW7 5BD UK; 65 Stockholm University, Department of Physical Geography, Stockholm, SE-106 91 Sweden; 66 Concordia University, Geography, Planning and Environment, Montreal, H3G 1M8 Canada; 67 Ca’ Foscari University of Venice, Department of Environmental Sciences, Informatics and Statistics, Venezia, 30172 Italy; 68 Herzen State Pedagogical University of Russia, Research Laboratory of the Environmental management, St. Petersburg, 191186 Russia; 69 Universitat de Barcelona, Departament de Biologia Evolutiva, Ecologia i Ciències Ambientals, Secció Ecologia, Barcelona, 08028 Spain; 70 University College Cork, Department of Geography, Cork, Ireland; 71 NORCE Norwegian Research Centre, LFI, Bergen, 5008 Norway; 72 US National Oceanic and Atmospheric Administration, National Centers for Environmental Information, Boulder, CO 80305 USA; 73 University of British Columbia, Department of Biology; Department of Earth, Environmental and Geographic Sciences, Kelowna, British Columbia V1V 1V7 Canada; 74 Landcare Research, Ecosystems and Conservation, Lincoln, 7640 New Zealand; 75 Chinese Academy of Sciences, Nanjing Institute of Geography and Limnology, Nanjing, 210008 China; 76 Institute of Archaeology and Ethnography, Russian Academy of Sciences, Siberian Branch, Novosibirsk 630090 Russia

**Keywords:** Palaeoclimate, Climate and Earth system modelling

## Abstract

A comprehensive database of paleoclimate records is needed to place recent warming into the longer-term context of natural climate variability. We present a global compilation of quality-controlled, published, temperature-sensitive proxy records extending back 12,000 years through the Holocene. Data were compiled from 679 sites where time series cover at least 4000 years, are resolved at sub-millennial scale (median spacing of 400 years or finer) and have at least one age control point every 3000 years, with cut-off values slackened in data-sparse regions. The data derive from lake sediment (51%), marine sediment (31%), peat (11%), glacier ice (3%), and other natural archives. The database contains 1319 records, including 157 from the Southern Hemisphere. The multi-proxy database comprises paleotemperature time series based on ecological assemblages, as well as biophysical and geochemical indicators that reflect mean annual or seasonal temperatures, as encoded in the database. This database can be used to reconstruct the spatiotemporal evolution of Holocene temperature at global to regional scales, and is publicly available in Linked Paleo Data (LiPD) format.

## Abstract (summary)

**Table:** 

Measurement(s)	climate
Technology Type(s)	digital curation
Factor Type(s)	temporal interval • geographic location • proxy type
Sample Characteristic - Environment	climate system
Sample Characteristic - Location	Earth (planet)

Machine-accessible metadata file describing the reported data: 10.6084/m9.figshare.11967924

## Background & Summary

Placing recent global warming in the context of natural climate variability requires the long-term perspective afforded by paleoclimate proxy records. Reconstructing past global climatic changes relies on a variety of evidence from a large number of well-distributed sites. Previous syntheses of Holocene temperature records have typically focused on specific time horizons (mostly 6000 years ago), and are based nearly entirely on pollen assemblages from terrestrial archives^1–4^, or are dominated by sea-surface temperatures^5^ near continental margins. Few global datasets have been compiled based on evidence from a wide variety of proxy types, including ecological, geochemical and biophysical evidence from both marine and terrestrial archives. Understanding of paleoclimate is enriched by a multi-proxy approach. Using multiple proxy types can help expand geographic coverage while enabling an assessment of inherent proxy biases. However, assembling a comprehensive database of continuous (time-series instead of time-slice) Holocene paleotemperature proxy records supported by a uniform suite of metadata descriptors across a wide variety of proxy data types is challenging^6^, and has not yet been attempted. In addition, a large portion of the data and metadata that form the basis of published paleoclimate studies have not been made available through public repositories, prior to this data synthesis.

This data descriptor presents version 1.0.0 of the Temperature 12k database (ref. ^7^, with additional supporting information and updates at: www.ncdc.noaa.gov/paleo/study/27330). It describes the methods used to assemble the database, including the criteria for record inclusion, and it describes each of the metadata fields that enable intelligent and automated reuse of the time-series data (Table 1). In addition, this data descriptor summarizes the major features of the records that comprise the database, including millennial-scale trends in Holocene temperature. The robustness of these major trends is explored by subdividing the dataset into various spatial, methodological, seasonal and other categories and visualizing the extent to which these data subsets represent the overall trends of the database both latitudinally, and through the past 12,000 years.

**Table 1:** Brief description of selected metadata fields used in the Temperature 12k database and shown in Suppl. Table 1.

Name (Suppl. Table 1)	LiPD variable name	Essential?	Description
Data Set Name	dataSetName	yes	collection of proxy data and metadata
**Site location**			
Site Name	geo_siteName	yes	site name or marine core identification
Country Ocean	geo_countryOcean	auto	auto-generated based on NASA GCMD convention
Latitude	geo_latitude	yes	site latitude in decimal degrees (negative for Southern Hemisphere)
Longitude	geo_longitude	yes	site longitude in decimal degrees (negative for Western Hemisphere)
Elevation	geo_elevation	yes	site elevation in meters (negative for below sea level)
**Source and attribution**			
Publication 1	pub1_doi	yes	DOI of primary bibliographic reference; typically the original study that describes the data and authored by the data generator
Publication 2	pub2_doi	no	DOI of secondary bibliographic reference; typically a refinement of the original study including a new temperature calculation based on the original data
Original Data Citation	originalDataUrl	yes	persistent URL or DOI of original archived data file; data not previously deposited in open-source repository = “this compilation”
Neotoma ID	neotomaDatasetId	no	DOI or data identifier for pollen assemblage and other data stored in Neotoma Paleoecology Database
**Proxy record**			
Archive Type	archiveType	yes	major category of archive type (e.g., lake sediment)
Proxy General	paleoData_proxyGeneral	yes	major category of proxy type used to group records for plotting figures
Proxy Type	paleoData_proxy	yes	proxy type (e.g., pollen)
Proxy Detail	paleoData_proxyDetail	yes	specific type of material analyzed; can include species
Calibration Method	calibration_method	yes	statistical method used for calibration; “NA” for non-calibrated proxy types
Calibration Seasonality	calibration_seasonality	no	specific months used for calibration
Paleo Data Notes	paleoData_notes	no	information from original study; specific methods or interpretation that can help users understand the appropriate use and limitations of the proxy record
**Climate interpretation**			
Variable Name	paleoData_variableName	yes	“temperature” for calibrated records; “temperatureComposite” for auto-averaged; other variable names for non-calibrated records (e.g., d18O)
Units	paleoData_units	yes	°C for calibrated records; other variable units for non-calibrated proxies (e.g., permil)
Datum	paleoData_datum	yes	“abs” = absolute temperature; “anom” = temperature relative to a reference (anomaly); “SMOW” or “PDB” for d18O
Climate Variable	climateInterpretation1_variable	yes	primary climate variable sensed by proxy (“T” for this data product)
Climate Variable Detail	climateInterpretation1_variableDetail	yes	what environmental temperature is represented by the sensor and at what level (e.g., water@surface)?
Seasonality	climateInterpretation1_seasonality	yes	season represented by the climate variable; specific month number when available (e.g., annual = 1 2 3 4 5 6 7 8 9 10 11 12), otherwise generalized term (e.g., summer)
Season General	climateInterpretation1_seasonalityGeneral	yes	“summerOnly” = warm season with no annual record at site; “summer+“ = warm season with annual record available at site; “winterOnly” and “winter+“ = as above but for cold season; “annual” = annual record
Direction	climateInterpretation1_direction	yes	“positive” for proxy values that increase with temperature; “negative” for values that decrease with temperature
**Time series**			
Min Year	minYear	auto	youngest proxy sample; auto-generated from the time series data
Max Year	maxYear	auto	oldest proxy sample; auto-generated from the time series data
Resolution	paleoData_medianRes12k	auto	median spacing between consecutive samples over the past 12 ka
Ages Per kyr	agesPerKyr	auto	number of 14C, U/Th, and tephra ages per 1000 years over the past 12 ka
**Quality control**			
In Compilation	paleoData_inCompilation	yes	“Temp12k” for records that meet the selection criteria; “Tverse” for temperature-sensitive records that do not meet the criteria
QC Certification	paleoData_QCCertification	yes	initials of co-author(s) who certified that record meets selection criteria and added QC comments
QC Comments	paleoData_QCnotes	no	interpretative comments that help future users reuse the data intelligently; time-series data that were digitized from a published figure; are flagged; justification for retaining records that do not meet the selection criteria are provided.
**Links to data**			
Link to LiPDverse	lipdverseLink	auto	URL link for viewing, downloading, and editing the underlying LiPD file

The data are useful for addressing a variety of paleoclimate research questions at global to regional scales. For example, they are needed to help understand how the ocean-atmosphere circulation has evolved along with past global climate changes. The database is designed for comparison with model-based simulations of climate, with the goal of evaluating model performance while gaining insights into the mechanisms and feedbacks associated with global climate change. Particular attention has been devoted to documenting the seasonality of temperature interpretations because climate forcing during the Holocene was dominated by orbitally controlled insolation changes that operated asymmetrically across the annual cycle^8^.

This database complements the PAGES 2k Consortium^9^ database of global paleotemperature records, which extends back 2000 years and is formatted similarly within the Linked PaleoData structure (LiPD^10^). The PAGES Iso2k^11^ database, which focuses on water isotope records over the past 2000 years, is also being developed in the LiPD structure. These higher-resolution (mostly annual) time series of 2000-year-long records provide a bridge between the overall lower-resolution time series of this database and the highly detailed, but relatively brief instrumental-based record of climate.

## Methods

The Temperature 12k database comprises paleotemperature records generated using a wide variety of techniques. It gathers data from previously published studies, each of which describes the methodologies used to generate the various data types, the scientific underpinnings of the techniques used to interpret the data in terms of past temperature change, and the important uncertainties. A major feature of this database is the integration of these complementary proxy data types and the harmonization of the metadata that describe them. The database is also quality-controlled; it comprises time-series data of relatively high resolution (sample spacing finer than 400 years; see below) and with a relatively well-established time scale, with the goal of creating a cohesive and uniform data product. This section describes the procedures used to assemble the database rather than the specific methods used to generate the individual data records that comprise the database.

### Procedures - data sources

The Temperature 12k database builds on several published paleoclimate data compilations, including one recently used to reconstruct Holocene temperature gradients across the Northern Hemisphere^12^, which itself drew from earlier compilations^5,13–15^. The majority of the pollen-based paleotemperature time series were obtained through the compilation of Marsicek *et al*.^16^. The majority of the marine records were gathered as part of the US-based Data Assimilation for Deep Time (DADT) project, and some were selected from the compilations of the German Climate Modeling Initiative (PalMod^17^). For these, we focused on the subset of proxy types that record (near) sea-surface temperature. In addition to culling data from previous paleoclimate data compilations, we searched the literature and public data repositories (PANGAEA and World Data Service for Paleoclimatology, NOAA) for appropriate records. The remainder of the datasets were obtained through either the supplements of publications, or from individual data generators, some of whom are co-authors on this data product. Several records were not available from the original data generators. To rescue such data, particularly where they fill geographic gaps, time series were digitized from the source publication (as noted in the metadata).

Most of the pollen-based paleotemperature time series, and data from marine and glacier-ice archives were previously available through public repositories. In contrast, of the 105 records based on chironomids and biomarkers from terrestrial deposits, only 37% were previously available through public repositories. In addition, this data product includes an expanded and harmonized suite of metadata for all of the records, including those that we obtained from data repositories. The database also includes the chronological data used to develop the age scale for the proxy time series. For a large portion of studies, this chronological information was not previously archived along with the proxy records *per se*.

Only data published in the peer-reviewed literature were considered. This restriction helps to assure that the data are high quality, intended for scientific reuse, and are supported by vetted, complete and often nuanced paleoclimate and geochronological interpretations.

### Procedures - selection criteria

The Temperature 12k database was designed to address research questions involving temperature change over the Holocene at regional to global scales. Thousands of studies have been published that attest to Holocene temperature changes. These records have widely variable temporal resolution, duration, and chronological control. In order to provide a consistent, quality controlled data product, we selected records that meet specific, quantitative criteria. These criteria were designed for relatively broad inclusion, while concentrating on the highest-resolution and well-dated records. The criteria were adjusted for selected sites to fill major geographic gaps, or for other reasons as justified by authors in the ‘*QC Comments*’ section of the metadata. The selection criteria were:

#### Temperature sensitivity

Only those proxy records that have a demonstrated relation with temperature were included. Because most of the paleotemperature time series are not sufficiently resolved temporally to meaningfully compare with instrumental-based observations, this demonstration of temperature sensitivity is typically based on accepted understanding of the proxy systems. The specific approach and the calibration data used for the resulting paleotemperature estimates are typically described in the original publication for each study site and are specified within the metadata for most records (‘*Calibration Method*’). That a proxy type is sensitive to temperature does not preclude its sensitivity to other environmental variables, such as moisture availability or salinity. The extent to which a proxy indicator is temperature-dependent can vary among sites and through time. Proxy records with multiple simultaneous interpretations, such as those interpreted as a combination of changes in precipitation amount and temperature, were generally excluded from the database, unless the authors of the original studies identified temperature as the primary control on the proxy.

Proxy data do not have to be converted to units of degrees to be useful indicators of past temperature.

This database includes 43 relative temperature indicators that are reported in their native proxy variables (e.g., δ^18^O of glacier ice). They are useful because (1) they attest to the timing and relative magnitude of change, which is sufficient for many statistical reconstruction methods, especially those that do not assume linearity between the proxy and the climate variable; (2) they are used in proxy-system modeling and in some cases can be compared directly to climate model output; and (3) they provide a more complete spatial coverage for the proxy network.

#### Duration and resolution

The database documents past temperature variability ranging in time-scale from multi-millennial trends to centennial excursions. In an effort to represent multi-millennial trends, while maintaining a relatively even temporal distribution of data coverage, we selected records that span a minimum continuous duration of 4000 years anytime within the past 12,000 years. To focus on records that can be used to resolve sub-millennial patterns, we selected time series with sample resolution (‘*Resolution*’) finer than 400 years (i.e., the median spacing between consecutive samples is less than 400 years over the past 12,000 years or over the full record length, if shorter). For some records, the selection cut-off values for duration and resolution were relaxed to improve the global coverage of the data network, especially in the Southern Hemisphere. These records are identified as such in ‘*QC Comments*.’

#### Chronological control

Age control is a fundamental variable underlying paleoclimate time series. We selected records that are supported by age control points with minimum spacing of 3000 years over the record duration or within the past 12,000 years. Records with gaps longer than 3000 years were accepted in data-poor regions, or for sequences that are supported by a relatively high frequency of ages (typically five or more over the Holocene). These exceptions are noted in ‘*QC comments*.’ The chronological control points for almost all records were copied from the original articles, downloaded from data repositories, or obtained from the data generators. Unless they were unavailable, this database includes the chronological data and metadata necessary to generate age-depth models for proxy data from sediment and speleothem archive types. In some cases, where the original chronology was obsolete, such as for those originally reported on a radiocarbon time scale, we generated a new age model using ‘Bacon’^18^ and added a note in the ‘*QC Comments*.*’* Chronological data include depth, raw radiometric or other types of age controls, errors, and associated corrections when available. Other metadata such as material type analysed and sample identifiers were also included when available.

## Data Records

This data descriptor presents version 1.0.0 of the Temperature 12k database (ref. ^7^, www.ncdc.noaa.gov/paleo/study/27330 and 10.25921/4RY2-G808).

### Metadata

The database includes a large variety of metadata to facilitate subsampling, analysis, and intelligent reuse (described briefly in Table 1). The metadata (Suppl. Table 1) include essential information, with one entry (row) for each proxy record (time series), with a large portion of sites (‘*Site Name*’) represented by more than one record. These are based on different proxy types (e.g., alkenone and Mg/Ca from the same marine sediment sequence), or they represent different seasons based on a single proxy type, usually pollen. This database is a subset of a larger compilation of paleoclimate datasets that are configured in the same format, including the PAGES 2k Consortium^9^ database. The 1319 records that comprise this data product are identified within this larger collection by ‘*In Compilation*’ = ‘Temp12k’. The final column in Suppl. Table 1, ‘*Link to LiPDverse*,*’* displays the URL that links from the metadata table to each dataset where the metadata and associated proxy time series and chronology data can be viewed and downloaded individually.

The specific metadata fields (Table 1; Suppl. Table 1) document information about the:

(1) site location, including: *‘Site Name*,’ ‘*Country Ocean*’ (based on NASA GCMD location keywords), latitude (‘*Latitude*’), longitude (‘*Longitude*’), and ‘*Elevation*.’ Geodetic data are in units of decimal degrees with respect to the WGS84 ellipsoid.

(2) bibliographic citations (DOI when available). ‘*Publication 1*’ typically refers to the original study that describes the data and was authored by the data generator, whereas ‘*Publication 2*’ typically refers to some refinement of the original study including subsequent paleotemperature analyses based on the original data. For most of the pollen records from North American and Europe, ‘*Publication 1*’ is the first of the citations listed for the dataset as referenced in the Neotoma Paleoecology Database (hereafter, “Neotoma”) and ‘*Publication 2*’ is the synthesis study of Marsicek *et al*.^16^, the most recent and comprehensive analysis of pollen data from this region.

(3) data source. ‘*Original Data Citation*’ is the data citation (persistent identifier) used to locate the proxy data and paleotemperature values in a long-term and publicly accessible repository. Data from online supplements of articles are often behind paywalls and therefore not public, and some have been superseded by versions that were subsequently modified and stored in data repositories. Data transferred to a public repository for the first time as part of this data product are listed as ‘*Original Data Citation’ =* ‘this compilation.’ Taxonomic assemblage data are beyond the scope of this paleoclimate-oriented database. For pollen, information is provided in the metadata to access the assemblage and other information in Neotoma. Namely, ‘*Neotoma ID’* is either the dataset identifier or the DOI for the landing page, which includes assemblage and other (meta) data for pollen records that are currently curated in Neotoma. For some marine microfossil records, *‘Original Data Citation’* is a link to the assemblage data stored at PANGAEA and WDS-NOAA Paleoclimatology.

(4) bio-physical indicator and method used to infer past temperature, including: ‘A*rchive Type*,’ ‘*Proxy General*’, and ‘*Proxy Type*,’ ‘*Proxy Detail*.’ The latter is particularly useful for proxy records that are based on isotope and geochemical analyses for which the specific sensor species or material type is essential information. ‘*Proxy General*’ is used to group proxy types to simplify plotting of figures. The ‘*Calibration Method*’ used to translate proxy data to temperature is stated for most calibrated proxy records, or is typically stated within the original publication. ‘*Paleo Data Notes*’ provides additional pertinent information about the proxy record, including its limitations as represented by the original study.

(5) climate interpretation. All of the records included in this data product are temperature sensitive (‘*Climate Variable*’ = ‘T’), and most are calibrated (‘*Variable Name*’ = ‘temperature’), either as absolute temperature (‘*Datum*’ = ‘abs’) or as a temperature anomaly (‘*Datum*’ = ‘anom’). ‘*Climate Variable Detail*’ provides further information about what environmental temperature is represented by the sensor (air, surface water, subsurface water). ‘*Variable Name*’ and ‘*Unit*s’ are = ‘temperature’ and ‘degC’ for the calibrated records in this data product. Some proxy records are related to, but not calibrated to temperature (‘*Variable Name*’ is not ‘temperature’ for these records). For these non-calibrated records, ‘*Variable Name*’ and ‘*Units*’ refer to the native proxy type, such as ‘d18O,’ which is expressed in units of ‘permil.’ ‘*Direction*’ applies to the non-calibrated proxy types. It is ‘positive’ for proxy values that increase with increasing temperature and ‘negative’ for values that are inversely related to temperature.

In some cases, authors of original studies presented alternative interpretations of temperature for a particular season based on a single proxy type. We selected the interpretation that the author of the original study deemed superior, or when ambiguous, authors of this data product made the selection based on rationale noted in the ‘*QC Comments*’ of the metadata. Preference was generally given to the highest resolution or most recent rendition of a proxy record. When there was no clear basis for selection, and different interpretations were based on the same proxy data (e.g., two different training sets were applied to the same assemblage data), the time series were combined, first by subtracting the record means to avoid artifacts related to combining time series of different lengths or number of samples, then by averaging to express temperatures as temperature anomalies (‘*Datum*’ = ‘anom’). These composited temperature records are designated, ‘*Variable Name*’ = ‘temperatureComposite’ and are noted in the ‘*QC comments*’. The paleotemperature time series used in the composites are retained in the database. Paleo temperatures from sites within the margins of former ice sheets were not corrected for the effect of isostatic rebound.

(6) time of year represented by the climate variable (‘*Seasonality*’). When available, specific months are listed according to the corresponding calendar-month number. Because of the wide variety of specific seasonalities included in the database, ‘*Season General*’ is used to generalize the seasonality as either annual, summer or winter. Several marine records represent transitional seasons; for these, spring was grouped with summer and fall with winter. Six or more months overlapping with June were categorized as annual. This field is also used to distinguish sites for which there are both summer and annual (= ‘summer+’ and ‘annual’) from sites where summer records are not paired with an annual counterpart (= ‘summerOnly’), with an equivalent formulation for ‘winter+’ and ‘winterOnly.’ This enables easy filtering of the database to select sites with either seasonal or annual time series, or both. When ‘*Variable Name*’ = ‘temperatureComposite,’ this time series is the average of winter and summer time series, which were calculated for this database to approximate annual values when no annual values are available, as indicated in the ‘*QC comments*.’ ‘*Calibration Season*’ (when available) specifies the exact month(s) to which the climate variable have been calibrated. For example, many Northern Hemisphere chironomid records are considered to represent summer temperature (‘*Seasonality*’ = ‘6 7 8’); however, they are usually calibrated against only July or August temperatures (‘*Calibration Season*’ = ‘7’ or ‘8’). For most records, ‘*Calibration Season*’ and ‘*Seasonality*’ are identical.

(7) chronology, including: youngest sample age (‘*Min Year*’), oldest sample age (‘*Max Year*’), and the median time-series resolution (‘*Resolution*’), which is calculated as the median difference between the modeled ages of consecutive proxy samples, extending back 12,000 years. To quantify the frequency of age-control points, ‘*Ages Per ky*r’ is calculated as the average number of radiocarbon (the vast majority of age types), tephra, and U/Th ages per 1000 years back to 12,000 years.

(8) quality control, including the initials of the author(s) (‘*QC Certification*’) of this data product who was (were) responsible for assuring that an individual record meets the selection criteria, or for justifying the inclusion of records that do not meet the criteria, and for entering additional comments to improve the reusability of the proxy record (‘*QC Comments*’).

(9) link to the data, including a browser-based interface for LiPD files (‘*Link to LiPDverse*’) with data-viewing and download capabilities (LiPD and.csv formats).

### Number and type of proxy records

**Fig. 1:**
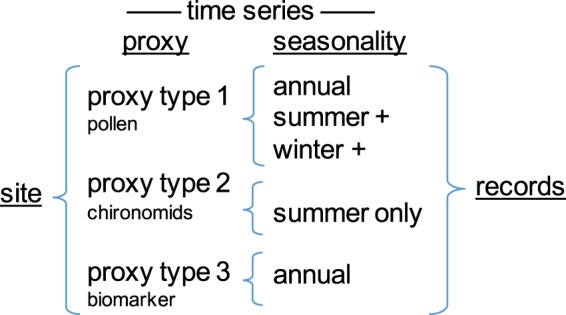
Nomenclature used in this data descriptor. This example illustrates one study *site* where time series are available for three *proxy types*, each of which is used to infer temperatures for different *seasonality*. This example shows 1 *site* where three *proxy time series* represent five *seasonality time series*, which we collectively and generally call, *records*.

In this data descriptor, the term “site” refers to a single location (or limited area) where various analyses were conducted to generate one or more “proxy time series” (Fig. 1). Each proxy time series is interpreted in terms of temperatures for one or more seasons, collectively and generally referred to as “records”.

**Fig. 2:**
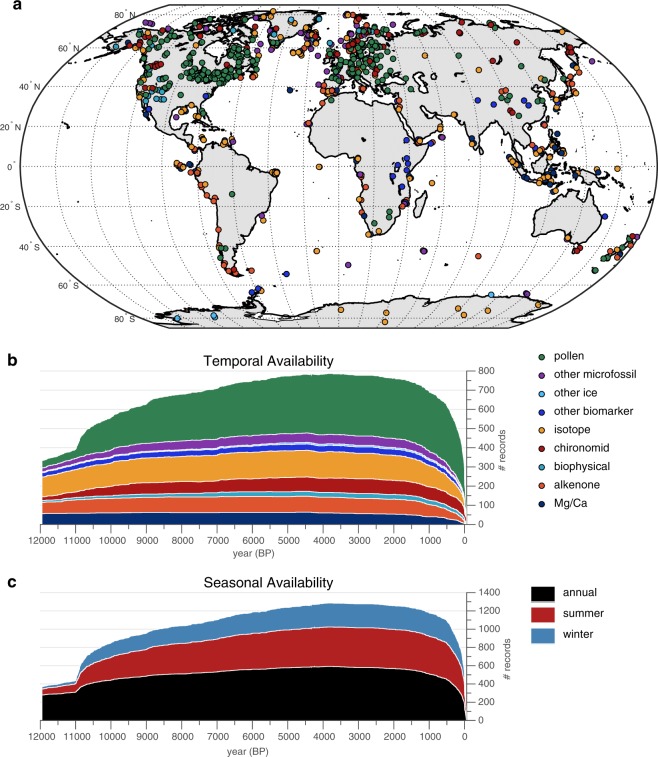
Spatiotemporal data availability of records in the Temperature 12k database (v. 1.0). (**a**) Geographical distribution of sites (n = 679) by proxy type, coded by color. (**b**) Temporal availability by proxy type, coded by colors as shown in (**a**). Proxy time series (Fig. 1) are represented by only one seasonal (or annual) record for each site, but all proxy types are counted (i.e., some sites include more than one proxy type for the same season; n = 816). Specific proxy types (Suppl. Table 1, ‘proxy’) are either grouped or treated separately (‘*Proxy General*’) depending on the number of records available. For example, *‘Proxy General*’ = ‘other microfossils’ includes ‘*Proxy Type*’ = dinocysts, foraminifera, diatoms and radiolaria, which together comprise a small number of records and were grouped and separated from the more numerous pollen and chironomid records. ‘*Proxy General*’ = ‘other biomarkers’ includes TEX_86_, GDGT, BNA15, LDI, but not alkenones, which are treated separately. ‘*Proxy General*’ = ‘other ice’ includes boreholes, bubble frequency, gas diffusion, melt-layer frequency, etc., but not isotopes. Refer to Suppl. Table 1 for details. (**c**) Temporal availability of records by seasons (Suppl. Table 1, ‘*Season General*’). Both annual and seasonal records from the same site are included (n = 1319).

The Temperature 12k database includes proxy time series from 470 terrestrial and 209 marine sites (Suppl. Table 2; Fig. 2). In total, this database comprises 1319 paleotemperature records. Multiple seasons are represented as different records at most sites, especially those based on pollen assemblages. In some cases, multiple records from the same site are based on different proxy types, most commonly planktic foraminifera δ^18^O, Mg/Ca, and alkenones from the same marine sediment core. The database includes 715 records from lake sediments, 359 records from marine sediments, and 245 from other terrestrial archives (e.g., glacier ice and speleothem). Alkenones and isotopes are the dominant sea-surface temperature proxies, whereas pollen and chironomids are the most common terrestrial temperature proxy types. In addition, the database includes paleotemperature evidence from a wide variety of other proxy types, such as assemblages of vegetation macrofossils from pack-rat middens, dinocysts from marine sediment, the composition of glycerol dialkyl glycerol tetraethers (GDGTs), abundance of chlorophyll from lake sediment, or the isotopic composition of pore ice in permafrost, to name a few.

The most frequent proxy type is pollen. Unlike other compilations of large-scale, pollen-based climate reconstructions, records in this database were screened for resolution and chronological control. In addition, the Temperature 12k database includes links (DOIs) to the primary pollen assemblage and additional data as curated in Neotoma. Most of the pollen-based paleotemperature time series from North America and Europe in this database are from the synthesis of Marsicek *et al*.^16^, which used the modern analogue technique to calculate paleotemperatures back to around 11 ka, and screened records using the PalaeoSig^19^ package (‘randomTF’ function with a 95% confidence level). Data from 209 of their 642 sites met our selection criteria. This database expands on the original data archive for ref. ^16^ (www.ncdc.noaa.gov/paleo-search/study/22992) by also including warmest-month temperatures based on the same pollen data and procedures, along with the mean annual temperatures. The ‘*Original Data Citation*’ for these extended records are therefore indicated as ‘this compilation’. In addition, this database includes the native-resolution time series for those datasets, whereas the original data archive features temperatures summarized in 100-year intervals.

The most frequent marine proxy types are δ^18^O, alkenones (U^K^’_37_), and Mg/Ca, including data from 135 out of 260 sites that were assimilated from the DADT project. For nearly all of these records, plus those based on TEX_86_, we generated paleotemperature time series by using the Bayesian calibration methods of Malevich *et al*.^20^, Tierney and Tingley^21,22^, and Tierney *et al*.^23^ respectively, with their published model parameters, as specified in ‘*Calibration Method*’. The original temperature time series are retained among the ancillary records in the larger collection of temperature-sensitive datasets (‘*In Compilation*’ = Tverse, see below) and are noted as such in the ‘*QC Comments*’ field. The ‘*Original Data Citation*’ for these datasets refer to the source of the underlying proxy data, some of which include calibrated temperatures from the original studies.

### Geographic coverage

**Fig. 3:**
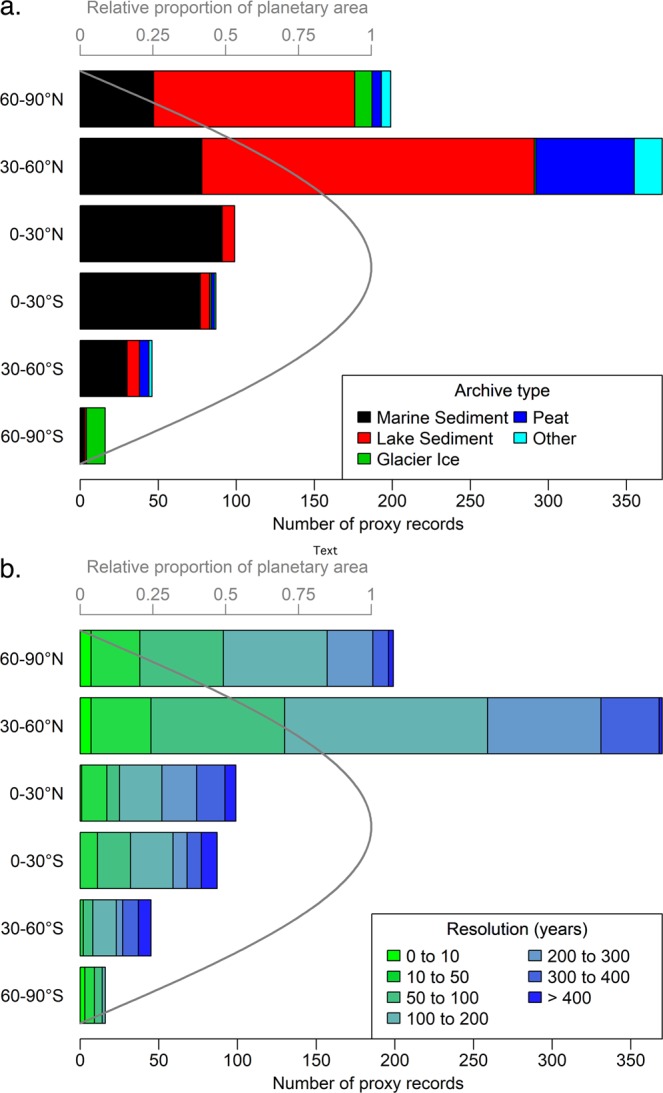
Latitudinal distribution of records. Frequency of records partitioned in 30° latitude bands according to their (**a**) archive type (Suppl. Table 1, ‘*Archive Type*’), and (**b**) temporal resolution (Suppl. Table 1, ‘*Resolution*’). Only one seasonal (or annual) record is counted for each proxy type from a site. Resolution calculated as the median spacing between consecutive proxy samples of each time series, back to 12,000 years.

The Temperature 12k database gathers paleotemperature data from every continent and ocean. The geographical distribution of the records, however, is uneven (Fig. 2). Latitudinally, 51% of the sites are located within the zone of 60–30°N, and only 16% are located in the Southern Hemisphere (Fig. 3). The spatial density of sites is comparatively high in North America and Europe and lower across the open ocean and tropical Africa. Data-poor regions reflect a combination of physical impossibility to obtain proxy records (e.g., low sediment accumulation rates in the open ocean and paucity of biogenic materials from extreme environments such as deserts), limited research attention and, in some cases, restricted field and data accessibility (e.g., Siberia).

### Record length and resolution

The temporal distribution of the time series is relatively uniform (Fig. 2b), with different proxy types having similar record lengths. The average record length within the Holocene is 9813 years for this database (n = 816, where only one season (or annual) is counted for each proxy from each site). All sites are represented by records that include at least some data between 8.5 and 3.5 ka. The number of records decreases over the last millennium, especially over the past century, largely because the surface sediment from lakes and oceans are watery and therefore difficult to recover intact, or because the climate interpretation of recently accumulated sediment has been compromised by human activities.

Most of the records (91%) extend back at least 6000 years, thereby encompassing the ‘6 ka time slice’, which is an on-going and long-standing target for paleoclimate modeling^24^. The temporal distribution of time series contrasts with paleotemperature data from the past 2000 years^9^, which are dominated by tree-ring records less than 500 years long. This underscores the complementary information afforded by this database when combined with the PAGES 2k Consortium^9^. The median record resolution (Suppl. Table 1, ‘*Resolution*’) of individual time series in the database is 164 years over the Holocene (n = 816, where only one season (or annual) is counted for each proxy from each site) (Fig. 3b). Overall, 15% of these records have 50-year resolution or finer, 39% have 51- to 150-year resolution, and 21% are coarser than 250-year resolution.

### Seasonality

Most of the sites (74%) include at least one record that was interpreted as mean annual temperature, or was calculated as the average of summer and winter values for this data product to represent annual temperature. Most of the sites (64%) have records that are interpreted by the original authors as representing summer temperature, and 39% of the sites include winter paleotemperature estimates. The temporal distribution of records by season through the Holocene (Fig. 2c) shows relatively uniform distribution of seasonal paleotemperature records.

### Chronology

The majority of records in this database are based on sedimentary sequences dated by radiocarbon, and their time series are calibrated to calendar years relative to 1950 CE (BP). Some of the age-depth models are supported by volcanic ash (tephra) whose ages are known, some are augmented by biostratigraphic markers (first-arrival datums for pollen records), and some have ^210^Pb profiles constraining the age of surface sediments. Speleothems are dated by U/Th methods, and some sequences are annually laminated (varves, ice, wood). For sequences that rely mainly on radiocarbon (n = 613), the average number of ages for records in this database is 1.0 per 1000 years, including the tephra ages. Recalculated age models based on a Bayesian modeling routine^18^ are available for many of the time series, including the marine records from the ongoing DADT project (records with ‘*Calibration Method*’ = ‘Bay...’), and McKay *et al*.’s Arctic Neoglacial study^25^. The age ensembles are made available as part of the expanded data package for this data product (see below).

## Technical Validation

Confirmation that the Temperature 12k dataset accurately represents the temperature at a site or globally would require knowing the actual temperature through time. This can only be determined for the period of instrumental temperature observations. Only a few of the records in this database include a sufficient number of inferred temperatures (samples) over the period of thermometer-based temperature observations for such a determination. In fact, the number of records that include data over the 20^th^ Century is less than any other century of the past 10,000 years. Instead, evidence that the records in the database reflect past temperature fluctuations at each site can typically be found in the original publications associated with each record (Suppl. Table 2, refs. ^26–566^). This procedure relies on expert knowledge as represented in peer-reviewed literature to guide the selection of proxy records. Records were not selected or weighted based on how well they correlate with an instrumental target. Expert knowledge can indeed yield a stronger proxy network for paleoclimate reconstructions than screening against instrumental data alone^567^.

The validation approach used here focuses on the robustness of the major trends that characterize this diverse dataset. For this, the database was subdivided and summarized statistically to evaluate the extent to which a common signal is represented by various categories of records, or whether the major features of the overall signal are strongly controlled by a particular subset of the records. We also test whether the spatial network of sites in this database is sufficient for representing large areas of the globe; for this, we focus on 30° latitudinal bands, following previous reconstructions of global temperature for the Holocene^5^ and the deglacial period^568^. This robustness testing is in addition to basic quality control procedures.

### Quality control procedures

The authors of this data product worked in four teams of proxy experts (marine, pollen, chironomid, and other terrestrial archives) to assemble and quality control the data and metadata. They used a web-based data viewer (LiPDverse.com) and other visualization tools to examine displays of the raw data and the metadata. They reviewed the primary literature to assure that the data met the selection criteria for this database. The metadata (Suppl. Table 1, ‘*QC Certification*’) lists the initials of the author(s) of this data descriptor who certified that each proxy record was translated accurately to the database, and that it was interpreted in the literature as related to temperature, and who, in some cases, added notes to help assure appropriate reuse of the data (‘*QC Comments*’).

In addition to the expert review, each record was analyzed using a series of automated tests to identify those with values that exceed thresholds defined by the physically realistic ranges for a variable. Records were flagged for follow-up if, for example, site coordinates exceeded −90° and 90° latitude, if marine site elevations were positive, if country names did not match those from Natural Earth Data (www.naturalearthdata.com) and ocean basins those from www.marineregions.org, if records contained duplicated ages, or if the temperature exceeded the range of −40 to 50 °C. The automated tests are based on the ‘pytest’ test framework as described in ref. ^569^.

### Robustness of major trends represented in different subsets of records

Composite time series were generated to characterize the major overall trends in the time series that comprise the dataset and to compare signals contained in various subsets of the database.

#### Compositing procedures

Our approach follows the data descriptor of the PAGES 2k Consortium^9^ temperature database. Briefly, all time series were standardized to z-scores, with a mean of zero and variance of 1 SD over the entire record length (except for the global composite, as specified below). Individual data points were binned by averaging the measurements within 500-year intervals (except for the high-resolution composite described below). We chose 500 years because it is compatible with the minimum 400-year-resolution selection criterion for records in this database, and because this broad bin size substantially mitigates the influence of age uncertainties. The binned records were then gridded spatially using an equal-area grid (4000 grids, each with area = 127,525 km^2^, following methods in ref. ^12^) to reduce the influence of clustered sites on the composites (except for the proxy-specific composites). The binned time series of various types (as specified below) inside each grid cell were averaged. The gridded data were also averaged into 30° latitudinal (zonal) bands to yield a single composite.

#### Composite uncertainties

The database contains a wide range of inter-record variance, which necessitates a large sample size to reconstruct regional or global temperature history. The uncertainty in the mean value of the composite at any time is related to both the number of records and their dispersion. To quantify this uncertainty, we used a bootstrap procedure that randomly sampled the proxy data network to generate an ensemble of composites from which the uncertainty was calculated. The procedure samples with replacement^570^ whereby the number of randomly selected records equals the total number of records, but individual record could be selected more than once in a given bootstrap sample. The procedure was repeated to generate 1000 datasets from which composites were calculated and the mean and 95% uncertainty intervals were derived. The uncertainty intervals generated by this procedure are widest where a composite is represented by the fewest records and where there is therefore less certainty in the mean value. This approach is different than representing uncertainty according to the dispersion among records, which may not fully reflect the confidence in the mean estimate.

#### Composite seasonality

Unless otherwise specified, the composites were based on annual records. Where annual records were not available, the summer or winter season was used (all sites where both are available were averaged to generate an annual paleotemperature time series). By combining annual and seasonal records, we assume that the temperature variability represented by the seasonal records correlates with mean annual temperature. PAGES 2k Consortium^9^ explored this assumption by correlating seasonal and mean-annual time series using a gridded temperature reanalysis product (HadCRUT4.2). They found that correlations are generally very high (r> 0.8) in the tropics, where the mean annual temperature range is small, and low in the extra-tropics, particularly over Northern Hemisphere continental interiors for summer, where the mean annual temperature range is large and dominated by winter synoptic variability. Therefore, summer records located in the tropics may be good surrogates for mean annual temperature, but less so for records located on Northern Hemisphere continents. Even for records that have been calibrated to annual temperature, the extent to which they accurately represent annual temperature depends on multiple factors, including assumptions that underlie the calibration procedures and the part of the seasonal cycle that influences each type of proxy sensor.

#### Comparison among proxy types

**Fig. 4:**
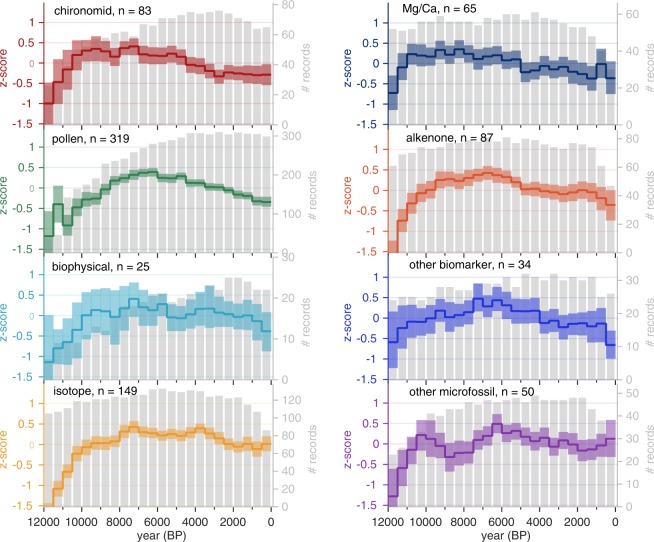
Major trends according to proxy type. Composites of normalized time series (standard deviation units; includes small portion of uncalibrated, relative proxy records) over the Holocene subdivided by major proxy types (Suppl. Table 1, ‘*Proxy Type*’). For sites with both annual and seasonal paleotemperature time series, only the annual time series was used (‘*Season General*’ = ‘annual’ OR ‘summerOnly’ OR ‘winterOnly’). Shading indicates 95% bootstrap confidence intervals with 1000 replicates. Gray bars indicate the number of records per bin. Specific proxy types are combined or treated separately depending on the number of records available (Suppl. Table 1, ‘*Proxy General*’ and ‘*Proxy Type*’; see Fig. 2 for explanation).

To evaluate the extent to which different proxy types carry a common overall signal, composites were calculated for each of the eight most common proxy types in the database (Fig. 4). No spatial gridding was applied prior to averaging for this comparison so as not to confound the direct comparison among proxy types. The composites all show warming trends during the early Holocene, some reaching peak warmth as early as around 10,000 years ago (e.g., chironomids), and others not until around 6000 years ago (e.g., pollen). By 6000 years ago, all of the composites show a cooling trend.

#### Comparison among seasonal and annual records

**Fig. 5:**
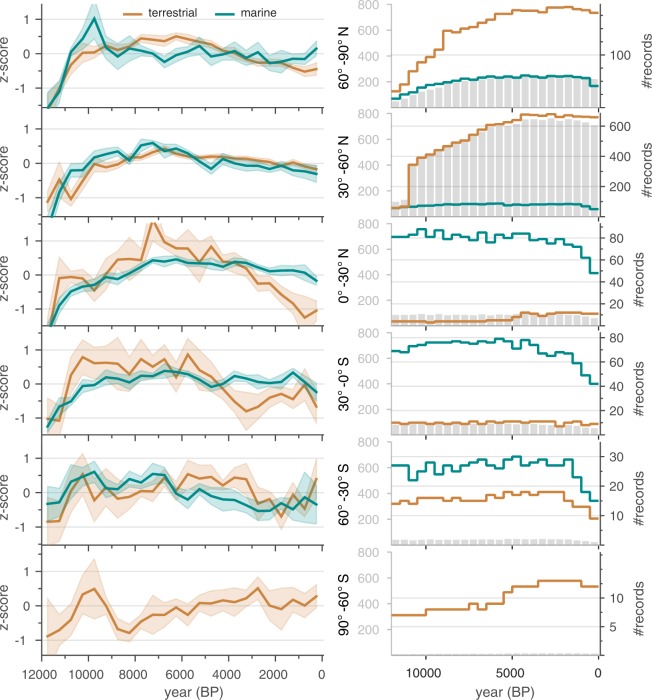
Comparison among summer, winter and annual records. Composites of normalized time series (standard deviation units; includes small portion of uncalibrated, relative proxy records) over the Holocene subdivided by season, binned at 500 years, averaged on an equal-area grid and then averaged over 30° latitude bands. For sites with both annual and seasonal paleotemperature time series, only the annual time series was used (Suppl. Table 1, ‘*Season General*’ = ‘annual’ OR ‘summerOnly’ OR ‘winterOnly’). Shading indicates 95% bootstrap confidence intervals with 1000 replicates. The column on the right shows the temporal availability for individual time series comprising the composites for each latitude band. Included are the total number of records per bin (gray bars) plotted on the same y-axis scale (left side, gray) across all latitudes, as well as the number of records by category (colored lines) plotted on a variably zoomed y-axis scale (right side).

Composites were calculated to evaluate the overall differences among records that represent annual, summer and winter temperatures (Fig. 5). For these composites, all of the proxy types were averaged within a grid cell and then across each latitudinal band for annual, summer, and winter time series. Nearly all composites show the general pattern of warming during the early Holocene then are either constant or cooling thereafter.

#### Comparison between marine and terrestrial records

**Fig. 6:**
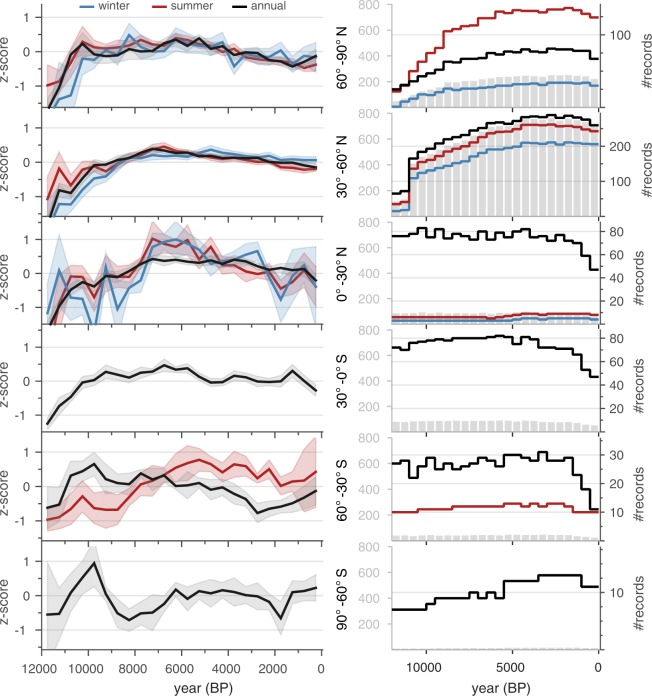
Comparison between records from terrestrial versus marine sites. Composite time series subdivided terrestrial versus marine archives. Marine sites include some terrestrially based proxy types, such as pollen and some biomarkers; these are represented by ‘*Climate Variable Detail*’ = ‘air@surface’ rather than ‘sea@surface’ (Suppl. Table 1). Symbols and procedures same as for Fig. 5.

Composites were calculated to evaluate the overall differences between marine and terrestrial sites (Fig. 6). For these composites, all of the proxy types were averaged within a grid for each of the two settings. The overall tendencies of the aggregated proxy time series from marine and terrestrial archives are similar. Nearly all composites warm during the early Holocene, then are either flat or cool thereafter, with a suggestion that tropical land records cooled more than for tropical sea surfaces.

#### Comparison between high- versus low-resolution records

**Fig. 7:**
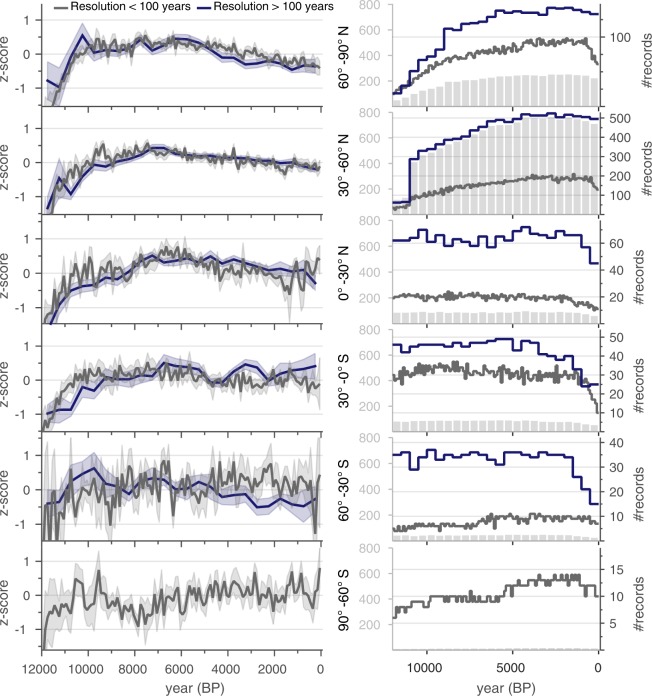
Comparison between low- and high-resolution records. Composite time series (standard deviation units; left side y-axis) for high-resolution versus low-resolution records binned at 100 and 500 year intervals, respectively. Cut-off between high and low resolution was set as 100 years (median difference between consecutive observations). Symbols and procedures as in Fig. 5.

Composites were calculated to evaluate the extent to which high- and low-resolution proxy records differ across all sites (Fig. 7). For this comparison, high-resolution records were chosen to have a median resolution finer than 100 years (Fig. 3b). The composites show that the millennial-scale trends of the high- and low-resolution records generally track each other. The high-resolution composites also exhibit greater variability than the low-resolution composites, as expected because the high-resolution composites are based on fewer records and because those records capture higher-frequency variability.

#### Global mean surface temperature, annual versus winter or summer

**Fig. 8:**
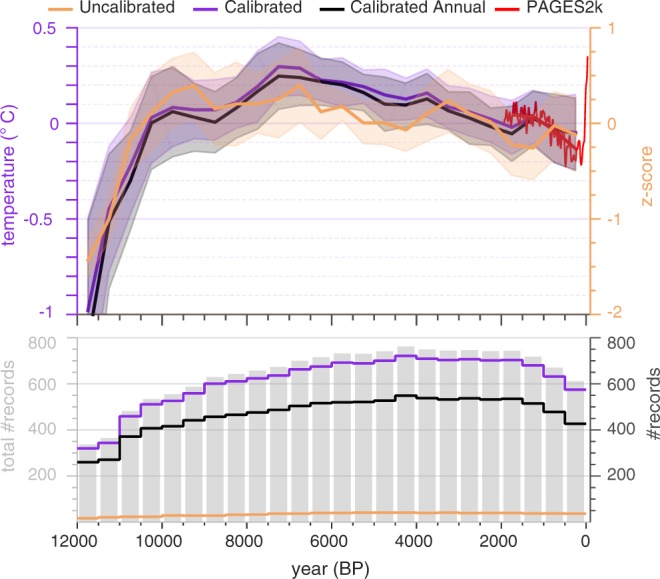
Comparison between calibrated versus uncalibrated records. Composite time series subdivided by records that are either calibrated to temperature (Suppl. Table 1, ‘*Units*’ = ‘degC’) or uncalibrated (n = 43; standard deviation units). Two calibrated composites are shown: black = annual records only (n = 612); purple = annual plus either summer or winter records for sites where annual records are not available (n = 816). The calibrated composites were placed on a temperature scale (left x-axis) by aligning the mean of each composite with the mean of the global temperature reconstruction from the PAGES 2k Consortium^571^, both over the period 500 and 1500 CE. Red = median of the PAGES 2k multi-method ensemble global mean surface temperature reconstruction binned at 500 years (bold red line) and with 30-year smoothing of annually resolved data (fine red line; data from www.ncdc.noaa.gov/paleo/study/21171). No instrumental data are shown. Symbols and procedures same as for Fig. 5.

A simple global composite of proxy records was calculated as the mean of six, 30° latitude averages, each weighted by the proportion of Earth’s area represented by that band (0.067, 0.183, and 0.25 for the high, middle, and low latitude bands, respectively) (Fig. 8). For this composite, records calibrated to temperature (°C) were averaged within grids, then across the latitude bands. To evaluate the effect of combining annual and seasonal records, two composites were calculated: one based on annual records only (n = 612) and one based on annual plus either summer or winter values for sites where annual values are not available (n = 816). The composites were registered to the temperature scale (left-side x-axis) by aligning the 500 to 1500 CE mean of the composite with the mean of the global temperature reconstruction from the same interval in the PAGES 2k Consortium^571^ multi-method median reconstruction. The variance of the Holocene temperature composites (all based on records in units of °C) were not scaled.

#### Comparison between calibrated and uncalibrated records (Fig. 8)

A composite of all relative proxy data (n = 43; those not calibrated to temperature in °C and not included in the other global composites in Fig. 8) was calculated for comparison with the calibrated proxy records. The composite of uncalibrated records comprises records from around the globe, but about half (53%) are based on water isotopes in polar ice or speleothems. While the general pattern of the composite based on uncalibrated proxies is similar to that of the calibrated proxies, minor differences are expected, especially considering the limited number of sites and polar bias of the uncalibrated records.

#### Zonal representativeness

**Fig. 9:**
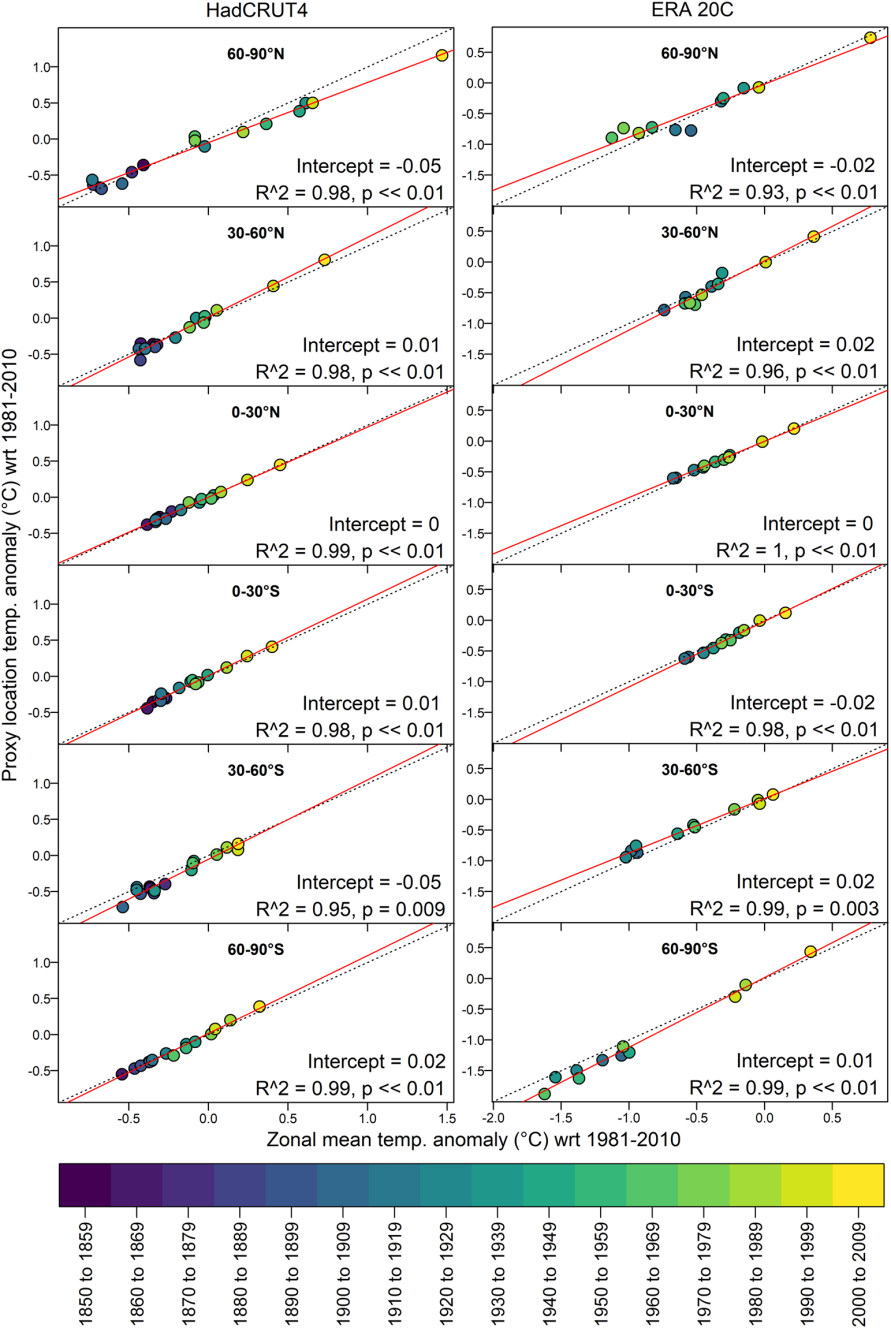
Zonal representativeness of the proxy network based on instrumental temperature. Scatterplots showing the relation between decadal mean temperature at the proxy locations versus the average of the entire 30° latitudinal zone using gridded instrumental-based temperature reanalysis products: (**a**) HadCRUT4 dataset^573,574^, (www.metoffice.gov.uk/hadobs/hadcrut4) and (**b**) ERA20C dataset^572^ (www.ecmwf.int/en/forecasts/datasets/reanalysis-datasets/era-20c). In the instrumental dataset, the mean temperature at the proxy locations explain between 93% and 100% of the temperature variance in the latitudinal bands. The spread in data represents the overall temperature trend over the 20th century.

**Fig. 10:**
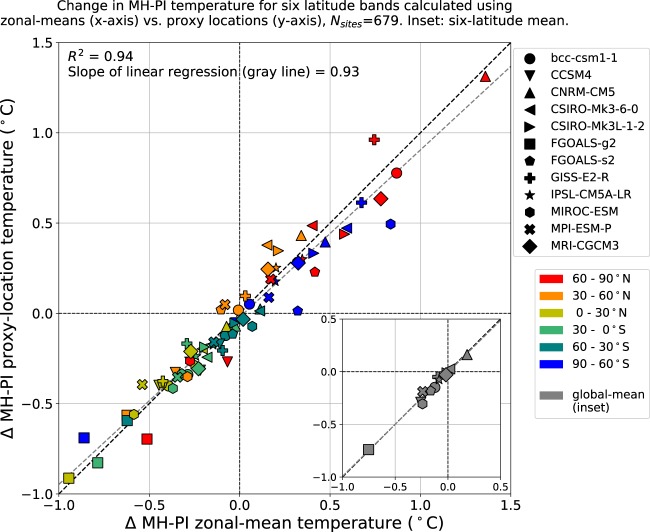
Zonal representativeness of the proxy network based on modelled temperature. Mid-Holocene minus preindustrial (MH − PI) annual temperature averaged for the proxy locations (y-axis) versus the annual temperature averaged over an entire 30°-wide latitudinal band (x-axis) from 12 PMIP3 climate models (symbols), shown for six latitudinal bands (colors). The proxy network sampled in the models captures the same mid-Holocene annual temperature anomalies as represented by the latitudinal averages. Global-mean values, calculated as the area-weighted mean of the six latitude bands, are shown in the inset. Linear regression of the global-mean values has an R^2^ of 0.98 and a slope of 0.99. PMIP3 model output is available at esgf-node.llnl.gov/projects/esgf-llnl.

We evaluated the extent to which the spatial network of proxy temperature sites accurately represents the latitudinal surface temperature distribution of the planet (Figs. 9 and 10). Gridded instrumental-based temperatures from two temperature reanalysis data products (ERA20C^572^ and HadCRUT^573,574^) were used to evaluate how well the proxy locations represent the mean temperature over each of the 30° latitudinal bands. Instrumental temperature data were binned to decadal resolution to better represent the long time-scales typically integrated by the proxy records. Grid cells corresponding to the locations of proxy records were then averaged and compared with the mean of the entire latitudinal band in which they are located. Temperatures at the proxy site locations are strongly correlated with the latitudinal average.

In addition to the instrumental data, we explored the representativeness of the proxy network using climate models (Fig. 10). Mid-Holocene (6 ka) and preindustrial (0 ka) simulations were analyzed in 12 general circulation models (GCMs) from the Paleoclimate Modelling Intercomparison Project Phase III (PMIP3; experimental design described in ref. ^575^) to assess how well the proxy network represents the temperature of the six latitudinal bands. Compared to the preindustrial period, mid-Holocene simulations are forced by altered astronomical parameters. Ice sheets had already melted to their preindustrial extents. The 12 GCMs are the same as those used in ref. ^12^ and were analysed using the same procedures. The change in mid-Holocene minus preindustrial temperatures was calculated for both the proxy locations and the latitudinal averages in models. The proxy locations generally explain 94% of the variance in the latitudinal averages of mid-Holocene minus pre-industrial changes across the multiple models. This number increases to 98% for global means computed from area-weighted means of the six latitude bands.

For these comparisons, the temperature of the grid cells nearest to each proxy-record site were averaged and compared with global and zonal mean temperatures within both the PMIP3 mid-Holocene simulations and the instrumental temperature data over the past 150–100 years. The comparisons show that, within the simulated and reanalysis datasets, the temperatures at the network sites correlate essentially one-to-one with the zonal and global temperatures. The comparison relies on model-smoothed and gridded data, however, and therefore assumes that each proxy location is indicative of the climate of the broader area. Although most proxy archives represent temperature over a relatively large area, we recognize that any proxy records that reflect variations over limited spatial scales (e.g., due to complex local topography) may degrade the network’s representation of zonal and global temperature.

## Usage Notes

### Uncertainties

This database is presently the most comprehensive compilation of a globally distributed, multi-proxy, quality-controlled Holocene paleotemperature time series. It includes records from a variety of terrestrial and marine proxy types, each based on their own principles and procedures, and all of them associated with an extensive literature. Background information about the proxy types and their underlying assumptions is available in textbooks devoted to the topic (e.g., ref. ^576^), and specific information about each proxy record is available through the original publications (Suppl. Table 2, refs. ^26–566^). In addition to the variety of proxy types, there are a variety of approaches used to characterize uncertainties related to paleotemperature interpretations. There are no standard procedures for either calculating or reporting uncertainties, with some procedures taking analytical uncertainty into account and others focusing on the conversion of proxy measurements into absolute temperature. In most cases, the original studies describe the uncertainties associated with each proxy climate record.

Among the important uncertainties to consider when using this database are those related to (1) calibration and proxy biases, (2) chronology, (3) spatiotemporal coverage. Depending on the scientific goal, uncertainties related to these and other factors can be substantial. On the other hand, some of these limitations are less important or not applicable, depending on the scientific question they are used to address. For example, calibration uncertainties are often large relative to the small amplitude of Holocene temperature changes, but these uncertainties become less important when investigating the relative magnitude of temperature changes rather than the absolute temperature.

#### Uncertainties related to calibration and proxy biases

Converting proxy data to paleotemperature estimates at a site-level relies on either (1) statistical procedures using observations of modern systems over the period of instrumentally based observations to infer the quantitative relation between the proxy value and temperature, or (2) transfer functions based on the correlation of biogeochemical properties or taxonomic assemblages over contemporary environmental gradients. These two approaches are referred to as “calibration in time” and “calibration in space,” respectively. Uncertainties reported for paleotemperatures based on calibration-in-time procedures are typically relatively small because the method is tuned to reconstruct temperature variability at a single site, although this characterization does not represent all potential uncertainties^577^. In contrast, uncertainties typically reported for calibration-in-space procedures are usually characterized by larger but more globally applicable uncertainties, as the proxy-environment relation is examined over a wide environmental gradient. In this database, author-reported temperature uncertainties are included when they were readily available, which was infrequently and based on a variety of approaches. Some studies characterize uncertainties based on measurement errors, some report apparent calibration uncertainty estimates, while others report rigorously cross-validated uncertainty values.

The wide-ranging approaches that have been used to characterize uncertainties involved in converting proxy values to the paleotemperatures hamper a meaningful and systematic representation of errors. For this reason, some paleoclimate syntheses aimed at large-scale reconstructions apply a single uncertainty estimate to each proxy type (e.g., ref. ^5^). Others apply a single statistical method to calculate uncertainties for published proxy data, often employing expanded calibration datasets and new statistical methods (e.g., ref. ^7^). In this database, most of the paleotemperature records based on pollen from North America and Europe are from the large-scale study of ref. ^16^ and most of the records based on marine sediments (other than those from microfossil assemblages) are calculated for this data product using the Bayesian procedures of refs. ^20–23^. For other proxy types, paleotemperature values and their uncertainties are based on multiple generations of analytical and calibration methods.

Biases can arise when proxy types that are most sensitive to summer conditions, common for biological indicators, are scaled to represent mean annual temperatures. Unlike paleotemperatures inferred from microfossil assemblages, other proxy types behave more like temperature sensors in a strict sense, meaning that their temperature signal is biased towards the season when the sensor is most abundant. This recording bias is not always explicitly addressed in the original publications and in these cases we used our expert knowledge to assign the seasonality and water depth (‘*Climate Variable Detail*’). However, our knowledge of the ecology of the proxy sensors is still limited and the assumption of a temporally constant recording bias may not always hold true.

#### Uncertainties related to chronology

The 3000-year-maximum spacing between age-control points was chosen as a relatively inclusive screening criterion. The accuracy of the time scales depends mainly on (1) how well the ages represent the true age of the proxy sensor itself, which can be an issue when, for example, a radiocarbon age on bulk sediment is used to represent the age of the proxy sensor (e.g., pollen), and (2) the uniformity of the accumulation rate of the archival medium (dominantly sediments in this database), which governs the accuracy of the interpolated ages of samples between control points^13^. Relative to these uncertainties, the analytical precision of the age determinations is typically minor. The inclusion of the primary chronology data and Bayesian-derived age ensembles for many time series in this database allows users to quantify and incorporate many, but not all, aspects of the age uncertainty into their own analyses.

#### Uncertainties related to spatiotemporal coverage

The suitability of the database to address different scientific questions depends on the particular spatial and temporal scale. Some regions are covered more densely than others, and the number of records available decreases as the demands for temporal resolution increase. At finer spatial and temporal scales, the number of records with sufficient resolution and geochronological control is limited and typically based on more recent studies. For example, only about 39% of the sites have records with resolution finer than 100 years.

### Future directions

The Temperature 12k database will form the foundation for new studies of Holocene global and regional surface temperature changes, and will help identify future research priorities. This machine-readable database includes multiple parameters for searching and filtering the proxy records, depending on the scientific objective. The database can be partitioned to study and compare different proxy types, seasons, and many other attributes. Previous proxy time series from a region can be used to compare with the results of future studies that report Holocene temperatures. Because the Temperature 12k database is relatively comprehensive, it can facilitate broader comparisons, both locally and regionally, than are commonly included in site-level studies. This avoids over-reliance on select records while providing an objective means to recognize aberrant or misinterpreted records through systematic comparison against the full body of other available records.

In addition to the time-series data in this database, an extensive literature describes complementary evidence for Holocene temperature fluctuations based on a variety of data types, including event-based observations (e.g., dated moraines). In the future, such information can be compared with the time-series data in this database to provide a more robust interpretation of past climate changes (e.g., across Alaska^578^, Arctic Canada and Greenland^579^, and North Atlantic and Fennoscandia^580^).

The simplistic time-series composites included in this data descriptor provide insights into the large-scale patterns of the proxy data, and provide a basis for comparison among different subsets of the diverse database. The area-weighted composite (Fig. 8) also serves as an initial approximation of the global mean surface temperature over the Holocene. This provides a foundation for a more in-depth analysis of temperature history based on this database, including a comparison of statistical methods for reconstructing global mean surface temperature.

The Temperature 12k database represents a concerted effort to generate a comprehensive product, but it is an ongoing effort, with new records continuing to be published. The database includes a large proportion of available published records that meet the selection criteria and that were recovered by the authors of this data product. Some published records that meet the criteria might have been inadvertently overlooked. Readers who know of missing datasets, especially those from data-poor regions, or who find errors in this version are asked to contact one of the primary authors so that future versions of the database will be more complete and accurate. Rather than issuing errata to this publication, errors and additions will be included in subsequent versions of the database and updated through the online data repository (see below).

### Ancillary data

#### Additional records within the temperature 12k datasets

In addition to the paleotemperature records used in the Temperature 12k compilation, the LiPD files also contain ancillary data from the same sites. For some sites, these include the native observations used to derive the paleotemperature values (e.g., Mg/Ca of foraminifera), or other data that are not directly related to climate but represent environmental changes at a study site that might be useful in interpreting the climatic significance of the record (e.g., sedimentary magnetic susceptibility). Some LiPD datasets include proxy data that are sensitive to climate variables other than temperature. These ancillary data are made available along with this data product, but have not been reviewed for accuracy, and some, including most of the pollen-based precipitation records, have not been vetted by peer review. Within the LiPD files, the records that comprise v.1.0.0 of the Temperature 12k database and were used to generate the figures in this data descriptor are discoverable in ref. ^7^ by filtering the metadata field, ‘*In Compilation*’ = ‘Temp12k’.

#### Temperature-sensitive records in addition to temperature 12k datasets

This database is a quality-controlled and curated subset of data culled from a larger collection of proxy climate data (LiPDverse.org), all structured in LiPD. Many temperature-sensitive proxy records that were gathered as part of this project do not meet the selection criteria for length, resolution, or age control as prescribed for this database. These data are likely useful for addressing scientific objectives that are outside the scope of this compilation. Many of the records have been reviewed by the authors of this data product, as indicated by their initials in ‘*QC Certification*,’ but most have not been reviewed for accuracy. Nonetheless, these data, including about 1110 records from over 560 sites are made available as part of the overall database of temperature-sensitive records. Within the LiPD files (ref. ^7^, www.ncdc.noaa.gov/paleo/study/27330) these are discoverable by filtering the metadata field, ‘*In Compilation*’ = ‘Tverse’.

#### Ensemble paleotemperature time series and age models

To characterize and quantify uncertainties associated with both chronologies and paleotemperatures, recent paleoclimate studies have featured ensembles of age models and proxy time series, most commonly derived from the posterior distribution of Bayesian statistical methods. Many of the datasets in the Temperature 12k database include age or temperature ensembles, or both. These are included in the expanded version of the Temperature 12k database (Table 2). Most of the age ensembles are associated with previous studies that recalculated age models based on the ‘Bacon’ modeling routine^18^. These are available for many of the time series, including the marine records from the DADT project (records with ‘*Calibration Method*’ = ‘Bay...’), and McKay *et al*.’s Arctic Neoglacial study^25^. In addition, we generated ensembles of paleotemperature time series for the marine records that were recalibrated for this database. These too are available in the LiPD files in the expanded version of the Temperature 12k database.

**Table 2:** Contents of files available on the landing page^*^ for Temperature 12k database.

File name	Contents
LoadData.md	Instructions for loading database (markdown-style text)
Temp12k_directory_LiPD_files	All LiPD files (not zipped)
Temp12k_directory_NOAA_files	All datasets that were deposited at WDS-NOAA Paleoclimatology for the first time as part of this compilation, NOAA template format
Temp12k_v1_0_0_LiPD.zip	Database in LiPD format
Temp12k_v1_0_0.mat	MATLAB-readable database
Temp12k_v1_0_0-ts.pkl	Python-readable database
Temp12k_v1_0_0.Rdata	R-readable database
Temp12k_with_ensembles_v1_0_0_LiPD.zip	Database in LiPD format including available age-model and marine-proxy ensembles
Temp12k_v1_essential_metadata.xlsx	Metadata for Temp12k v.1.0.0 (same as Suppl. Table 1)
Temp12k_v1_record_list.xlsx	Temperature 12k records listed alphabetically
Temp12k_Composite_timeseries.zip	Composite time series shown in Figs. 5–8

*www.ncdc.noaa.gov/paleo/study/27330, DOI: 10.25921/4RY2-G808.

### Database format and operability

The site-level proxy and geochronology data and metadata are formatted in the Linked Paleo Data (LiPD) structure. The LiPD framework comprises JSON formatted, standardized files that are machine-readable in multiple programming languages for querying and data extraction^10^. The hierarchical structure allows explicit descriptions at any level and aspect of the database, thereby providing a flexible structure that can accommodate a variety of data and metadata types. The LiPD files comprising this database have also been excerpted and translated into the format of the World Data Service (WDS) NOAA Paleoclimatology where they are archived for long-term reuse.

### WDS-NOAA-Paleoclimatology Landing page contents

The entire database is available in LiPD format through WDS-NOAA Paleoclimatology (www.ncdc.noaa.gov/paleo/study/27330; 10.25921/4RY2-G808), with serializations for MATLAB, Python, and R. Any updates to the database will be posted at WDS-NOAA Paleoclimatology. The landing page links to digital versions of the Temperature 12k database, including the metadata in Suppl. Table 1, as well as to the composite time series generated by this study (Table 2).

### Versioning scheme

The database versioning scheme for this data product follows the one proposed by McKay and Emile-Geay^10^ and used for the PAGES 2k Consortium^9^ temperature database. The version number is in the form C1.C2.C3, where C1 is an integer associated with a publication, C2 is a counter updated every time a record is added or removed, and C3 is a counter updated every time a modification is made to the data or metadata in an individual record. The dataset published here is thus v1.0.0 of the Temperature 12k database. Future versions, along with a change log that specifies the modifications associated with each version, will be posted on the WDS-NOAA Paleoclimatology landing page. This versioning applies only to the temperature-sensitive records marked as ‘*In Compilation*’ = Temp12k; changes to ancillary time series are not tracked.

## Supplementary information

Supplementary Table 1

Supplementary Table 2

## Data Availability

Code for working with the LiPD data files, including basic functionality in three programming languages, is available on GitHub (https://github.com/nickmckay/LiPD-utilities). MATLAB code used to map site locations (Fig. 2) and to compute composites (Figs. 4–8) is available at https://github.com/nickmckay/Temperature12k under an MIT license^581^.

## References

[1] Mauri A, Davis BAS, Collins PM, Kaplan JO (2015). The climate of Europe during the Holocene: a gridded pollen-based reconstruction and its multiproxy evaluation. Quat. Sci. Rev..

[2] Harrison SP (2014). Climate model benchmarking with glacial and mid-Holocene climates. Clim. Dyn..

[3] Bartlein PJ (2011). Pollen-based continental climate reconstructions at 6 and 21 ka: A global synthesis. Clim. Dyn..

[4] Viau, A. E., Gajewski, K., Sawada, M. C. & Fines, P. Millennial-scale temperature variations in North America during the Holocene. *J*. *Geophys*. *Res*. *Atmospheres* **111** (2006).

[5] Marcott SA, Shakun JD, Clark PU, Mix AC (2013). A reconstruction of regional and global temperature for the past 11,300 years. Science.

[6] Kaufman, D. *Bookkeeping or science: what’s behind a paleo data compilation*. http://blogs.nature.com/soapboxscience/2017/07/11/bookkeeping-or-science-whats-behind-a-paleo-data-compilation (2017).

[7] Kaufman DS (2019). figshare.

[8] Renssen H, Seppä H, Crosta X, Goosse H, Roche DM (2012). Global characterization of the Holocene Thermal Maximum. Quat. Sci. Rev..

[9] PAGES 2k Consortium (2017). A global multiproxy database for temperature reconstructions of the Common Era. Sci. Data.

[10] McKay NP, Emile-Geay J (2016). Technical note: The Linked Paleo Data framework – a common tongue for paleoclimatology. Clim. Past.

[11] Konecky, B. L. *et al*. The Iso2k Database: A global compilation of paleo-δ^18^O and δ^2^H records to aid understanding of Common Era climate. *Earth Sys*. *Sci*. *Data Disc*., 10.5194/essd-2020-5 (2020).

[12] Routson CC (2019). Mid-latitude net precipitation decreased with Arctic warming during the Holocene. Nature.

[13] Sundqvist HS (2014). Arctic Holocene proxy climate database: new approaches to assessing geochronological accuracy and encoding climate variables. Clim. Past.

[14] Chen F (2008). Holocene moisture evolution in arid central Asia and its out-of-phase relationship with Asian monsoon history. Quat. Sci. Rev..

[15] Wanner H, Solomina O, Grosjean M, Ritz SP, Jetel M (2011). Structure and origin of Holocene cold events. Quat. Sci. Rev..

[16] Marsicek J, Shuman BN, Bartlein PJ, Shafer SL, Brewer S (2018). Reconciling divergent trends and millennial variations in Holocene temperatures. Nature.

[17] Jonkers, L. *et al*. Integrating palaeoclimate time series with rich metadata for uncertainty modelling: Strategy and documentation of the PALMOD 130k marine palaeoclimate data synthesis. *Earth Sys*. *Sci*. *Data Disc*. 10.5194/essd-2019-223 (2020).

[18] Blaauw M, Christen JA (2011). Flexible paleoclimate age-depth models using an autoregressive gamma process. Bayesian Anal..

[19] Trachsel M, Telford RJ (2016). Technical note: Estimating unbiased transfer-function performances in spatially structured environments. Clim. Past.

[20] Malevich SB, Vetter L, Tierney JE (2019). Global core top calibration of δ^18^O in planktic foraminifera to sea surface temperature. Paleoceanogr. Paleoclimatology.

[21] Tierney JE, Tingley MP (2018). BAYSPLINE: A new calibration for the alkenone paleothermometer. Paleoceanogr. Paleoclimatology.

[22] Tierney JE, Tingley MP (2014). A Bayesian, spatially-varying calibration model for the TEX86 proxy. Geochim. Cosmochim. Acta.

[23] Tierney JE, Malevich SB, Gray W, Vetter L, Thirumalai K (2019). Bayesian calibration of the Mg/Ca paleothermometer in planktic foraminifera. EarthArXiv Prepr..

[24] Otto-Bliesner BL (2017). The PMIP4 contribution to CMIP6 – Part 2: Two interglacials, scientific objective and experimental design for Holocene and Last Interglacial simulations. Geosci. Model Dev..

[25] McKay NP, Kaufman DS, Routson CC, Erb MP, Zander PD (2018). The onset and rate of Holocene neoglacial cooling in the Arctic. Geophys. Res. Lett..

[26] Godad SP, Naidu PD, Malmgren BA (2011). Sea surface temperature changes during May and August in the western Arabian Sea over the last 22kyr: Implications as to shifting of the upwelling season. Mar. Micropaleontol..

[27] Martrat B, Jimenez-Amat P, Zahn R, Grimalt JO (2014). Similarities and dissimilarities between the last two deglaciations and interglaciations in the North Atlantic region. Quat. Sci. Rev..

[28] Herbert, T.D. & Schuffert, J.D. Alkenone unsaturation estimates of sea-surface temperatures at site 1002 over a full glacial cycle. in: *Proc. ODP, Sci. Results* **165** (eds. Leckie, R.M., Sigurdsson, H., Acton, G.D., Draper, G.) 1–9 (College Station, TX, 2000).

[29] Ledu, D., Rochon, A., de Vernal, A., Barletta, F. & St-Onge, G. Holocene sea ice history and climate variability along the main axis of the Northwest Passage, Canadian Arctic. *Paleoceanography* **25**, PA2213 (2010).

[30] Pellatt MG, Smith MJ, Mathewes RW, Walker IR, Palmer SL (2000). Holocene treeline and climate change in the subalpine zone near Stoyoma Mountain, Cascade Mountains, southwestern British Columbia, Canada. Arct. Antarct. Alp. Res..

[31] Kennett DJ, Kennett JP, Erlandson JM, Cannariato KG (2007). Human responses to Middle Holocene climate change on California’s Channel Islands. Quat. Sci. Rev..

[32] Sun, Y., Oppo, D. W., Xiang, R., Liu, W. & Gao, S. Last deglaciation in the Okinawa Trough: Subtropical northwest Pacific link to Northern Hemisphere and tropical climate. *Paleoceanography* **20**, PA4005 (2005).

[33] Cole KL, Liu G-W (1994). Holocene paleoecology of an estuary on Santa Rosa Island, California. Quat. Res..

[34] Jara IA (2015). Pollen-climate reconstruction from northern South Island, New Zealand (41°S), reveals varying high- and low-latitude teleconnections over the last 16 000 years. J. Quat. Sci..

[35] Lecavalier BS (2017). High Arctic Holocene temperature record from the Agassiz ice cap and Greenland ice sheet evolution. Proc. Natl. Acad. Sci..

[36] Nilsson, T. Standard pollen diagramme und C14 datiengen aus dem Ageroeds mosse in mittleren schonen. *Lunds Univ*. *Arsskrift NF 2* **59**, 1–57 (1964).

[37] Wilmshurst JM, McGlone MS, Leathwick JR, Newnham RM (2007). A pre-deforestation pollen-climate calibration model for New Zealand and quantitative temperature reconstructions for the past 18 000 years BP. J. Quat. Sci..

[38] Heinrichs ML, Peglar SM, Bigler C, Birks HJB (2008). A multi-proxy palaeoecological study of Alanen Laanijärvi, a boreal-forest lake in Swedish Lapland. Boreas.

[39] Birks HH (1975). Studies in the vegetational history of Scotland. IV. Pine stumps in Scottish blanket peats. Philos. Trans. R. Soc. B Biol. Sci..

[40] Welten M (1982). Vegetationsgeschichtliche Untersuchungen in den westlichen Schweizer Alpen: Bern-Wallis. Denkschr. Schweiz. Naturforschenden Ges..

[41] Mariscal B (1993). Variacion de la vegetacion Holocena (4300-280 BP) de Cantabria a traves del analisis polinico de la turbera del Alsa. Estud. Geológicos.

[42] Zatykó, C., Juhász, I. & Sümegi, P., eds. (2007) Environmental archaeology in Transdanubia. *Varia archaeologica Hungarica* **20** (2007).

[43] De Valk, E. J. Late Holocene and present vegetation of the Kastelberg (Vosges, France). (University of Utrecht, 1981).

[44] von Grafenstein U, Erlenkeuser H, Müller J, Trimborn P, Alefs J (1996). A 200 year mid-European air temperature record preserved in lake sediments: An extension of the δ18Op-air temperature relation into the past. Geochim. Cosmochim. Acta.

[45] van der Bilt WGM (2018). Alkenone-based reconstructions reveal four-phase Holocene temperature evolution for High Arctic Svalbard. Quat. Sci. Rev..

[46] Szeicz JM, MacDonald GM, Duk-Rodkin A (1995). Late Quaternary vegetation history of the central Mackenzie Mountains, Northwest Territories, Canada. Palaeogeogr. Palaeoclimatol. Palaeoecol..

[47] Sarmaja-Korjonen K, Seppä H (2007). Abrupt and consistent responses of aquatic and terrestrial ecosystems to the 8200 cal. yr cold event: A lacustrine record from Lake Arapisto, Finland. The Holocene.

[48] Gauthier, R. *Histoire de la colonisation vegetale postglaciaire des Monteregiennes: Deux sites du mont Saint-Bruno*. (Universite de Montreal, 1981).

[49] Seppä H, Bjune AE, Telford RJ, Birks HJB, Veski S (2009). Last nine-thousand years of temperature variability in Northern Europe. Clim. Past.

[50] Li X, Wang M, Zhang Y, Lei L, Hou J (2017). Holocene climatic and environmental change on the western Tibetan Plateau revealed by glycerol dialkyl glycerol tetraethers and leaf wax deuterium-to-hydrogen ratios at Aweng Co. Quat. Res..

[51] Castañeda IS, Smith LM, Kristjánsdóttir GB, Andrews JT (2004). Temporal changes in Holocene δ^18^O records from the northwest and central North Iceland Shelf. J. Quat. Sci..

[52] Garnaud S (2003). Holocene to modern fine-grained sedimentation on a macrotidal shoreface-to-inner-shelf setting (eastern Bay of the Seine, France). Mar. Geol..

[53] Gajewski K, Mott RJ, Ritchie JC, Hadden K (2000). Holocene vegetation history of Banks Island, Northwest Territories, Canada. Can. J. Bot..

[54] Tarrats P (2018). Chironomid-inferred Holocene temperature reconstruction in Basa de la Mora Lake (Central Pyrenees). The Holocene.

[55] Voeltzel, D. *Recherches pollenanalytiques sur la vegetation holocene de la plaine alluviale de l’estuaire de la Loire et des coteaux environnants*. (Université Paul Cézanne, 1987).

[56] Bennett KD (1987). Holocene history of forest trees in southern Ontario. Can. J. Bot..

[57] Barnosky CW (1985). Late Quaternary vegetation near Battle Ground Lake, southern Puget Trough, Washington. Geol. Soc. Am. Bull..

[58] Peros M, Gajewski K, Paull T, Ravindra R, Podritske B (2010). Multi-proxy record of postglacial environmental change, south-central Melville Island, Northwest Territories, Canada. Quat. Res..

[59] Ritchie JC (1976). The late-Quaternary vegetational history of the western interior of Canada. Can. J. Bot..

[60] Shulija, K. S., Lujanas, V. J., Kibilda, Z. A., Banys, J. J. & Genutiene, I. K. Stratigraphy and chronology of lacustrine and bog deposits of the Bebrukas Lake hollow. *Tr*. *Instituta Geol*. *Vilnius* **5** (1967).

[61] Petersen KL (1985). Palynology in Montezuma County, southwestern Colorado: the local history of pinyon pine (*Pinus edulis*). ASSP Contrib. Ser..

[62] Ilyashuk EA, Ilyashuk BP, Hammarlund D, Larocque I (2005). Holocene climatic and environmental changes inferred from midge records (Diptera: Chironomidae, Chaoboridae, Ceratopogonidae) at Lake Berkut, southern Kola Peninsula, Russia. The Holocene.

[63] Whitehead DR (1979). Late-glacial and postglacial vegetational history of the Berkshires, western Massachusetts. Quat. Res..

[64] Gajewski K, Garralla S (1992). Holocene vegetation histories from three sites in the tundra of northwestern Quebec, Canada. Arct. Alp. Res..

[65] Barber K, Brown A, Langdon P, Hughes P (2013). Comparing and cross-validating lake and bog palaeoclimatic records: a review and a new 5,000 year chironomid-inferred temperature record from northern England. J. Paleolimnol..

[66] Jacobson, G. L., Jr. *A palynological study of the history and ecology of white pine in Minnesota*. (University of Minnesota, 1975).

[67] Noryskiewicz, B. Zmiany szaty roslinnej okolic Jeziora Biskupinskiego pod wplywem czynnikow naturalnych i antropogenicznych w poznym glacjale i holocenie [Changes in vegetation of the Biskupin (Biskupinskie) lake area during the Late-Glacial and the Holocene, caused by natural and anthropogenic factors]. 147–180 (1995).

[68] Linsley BK, Rosenthal Y, Oppo DW (2010). Holocene evolution of the Indonesian throughflow and the western Pacific warm pool. Nat. Geosci..

[69] Brooks SJ (2006). Fossil midges (Diptera: Chironomidae) as palaeoclimatic indicators for the Eurasian region. Quat. Sci. Rev..

[70] Binka K, Madeyska T, Marciniak B, Seroczynska K, Wieckowski K (1988). Bledowo Lake (central Poland): History of vegetation and lake development during the last 12 kyr. Bull Acad Pol. Sci.

[71] Marsicek JP, Shuman B, Brewer S, Foster DR, Oswald WW (2013). Moisture and temperature changes associated with the mid-Holocene *Tsuga* decline in the northeastern United States. Quat. Sci. Rev..

[72] Markgraf V (1969). Moorkundliche und vegetationsgeschichtliche Untersuchungen an einem Moorsee an der Waldgrenze im Wallis. Bot. Jahrbuecher.

[73] White JM, Mathewes RW (1986). Postglacial vegetation and climatic change in the upper Peace River district, Alberta. Can. J. Bot..

[74] Lamb HF (1985). Palynological evidence for postglacial change in the position of tree limit in Labrador. Ecol. Monogr..

[75] Mott RJ (1977). Late-Pleistocene and Holocene palynology in southeastern Québec. Géographie Phys. Quat..

[76] von Gunten L, D’Andrea WJ, Bradley RS, Huang Y (2012). Proxy-to-proxy calibration: Increasing the temporal resolution of quantitative climate reconstructions. Sci. Rep..

[77] Roesch M (1989). Pollenprofil Breitnau-Neuhof: Zum zeitlichen Verlauf der holozanen Vegetationsentwicklung im sudlichen Schwarzwald. Carolinea.

[78] Velle G, Brooks SJ, Birks HJB, Willassen E (2005). Chironomids as a tool for inferring Holocene climate: An assessment based on six sites in southern Scandinavia. Quat. Sci. Rev..

[79] Cacho I (2001). Variability of the western Mediterranean Sea surface temperature during the last 25,000 years and its connection with the Northern Hemisphere climatic changes. Paleoceanography.

[80] Weirich J, Bortenschlager S (1980). Beitraege zur Vegetationsgeschichte Tirols III: Stubaier Alpen - Zillertaler Alpen. Ber Nat-Med Ver. Innsbr..

[81] Niemann H (2012). Bacterial GDGTs in Holocene sediments and catchment soils of a high alpine lake: application of the MBT/CBT-paleothermometer. Clim. Past.

[82] Brubaker LB (1975). Postglacial forest patterns associated with till and outwash in Northcentral Upper Michigan. Quat. Res..

[83] Cwynar LC, Spear RW (1995). Paleovegetation and paleoclimatic changes in the Yukon at 6 ka BP. Géographie Phys. Quat..

[84] Talma AS, Vogel JC (1992). Late Quaternary paleotemperatures derived from a speleothem from Cango Caves, Cape Province, South Africa. Quat. Res..

[85] Kim J-H (2007). Impacts of the North Atlantic gyre circulation on Holocene climate off northwest Africa. Geology.

[86] Jetté H, Richard PJH (1992). Contribution à l’histoire postglaciaire de la végétation en Gaspésie méridionale, Québec. Géographie Phys. Quat..

[87] Upiter LM (2014). Middle to late Holocene chironomid-inferred July temperatures for the central Northwest Territories, Canada. J. Paleolimnol..

[88] Barnosky CW (1985). Late Quaternary vegetation in the southwestern Columbia Basin, Washington. Quat. Res..

[89] Maher LJ (1963). Pollen analyses of surface materials from the southern San Juan Mountains, Colorado. Geol. Soc. Am. Bull..

[90] Gibb OT, Steinhauer S, Fréchette B, de Vernal A, Hillaire-Marcel C (2015). Diachronous evolution of sea surface conditions in the Labrador Sea and Baffin Bay since the last deglaciation. The Holocene.

[91] Johnsen SJ, Dansgaard W, Clausen HB, Langway CC (1972). Oxygen isotope profiles through the Antarctic and Greenland Ice Sheets. Nature.

[92] Axford Y (2011). Chironomids record terrestrial temperature changes throughout Arctic interglacials of the past 200,000 yr. Geol. Soc. Am. Bull..

[93] Sinninghe Damsté JS, Ossebaar J, Schouten S, Verschuren D (2012). Distribution of tetraether lipids in the 25-ka sedimentary record of Lake Challa: Extracting reliable TEX86 and MBT/CBT palaeotemperatures from an equatorial African lake. Quat. Sci. Rev..

[94] Li J (2018). Quantitative Holocene climatic reconstructions for the lower Yangtze region of China. Clim. Dyn..

[95] Solovieva N, Tarasov PE, MacDonald G (2005). Quantitative reconstruction of Holocene climate from the Chuna Lake pollen record, Kola Peninsula, northwest Russia. The Holocene.

[96] Caniupán M (2014). Holocene sea-surface temperature variability in the Chilean fjord region. Quat. Res..

[97] de Beaulieu, J. L. *Contribution pollenanalytique a l’histoire tardiglaciaire et Holocene de la vegetation des Alpes meridionales francaises*. (Universite d’Aix-Marseille, 1977).

[98] Baker RG, Maher LJ, Chumbley CA, Van Zant KL (1992). Patterns of Holocene environmental change in the Midwestern United States. Quat. Res..

[99] Bailey, R. E. *Late- and postglacial environmental changes in northwestern Indiana*. (Indiana University, 1972).

[100] Hussey, T. C. *A 20*,*000-year history of vegetation and climate at Clear Pond*, *northeastern South Carolina*. (University of Maine, 1993).

[101] Cheung M-C, Zong Y, Zheng Z, Liu Z, Aitchison JC (2017). Holocene temperature and precipitation variability on the central Tibetan Plateau revealed by multiple palaeo-climatic proxy records from an alpine wetland sequence. The Holocene.

[102] King, G. A. *Deglaciation and vegetation history of western Labrador and adjacent Quebec*. (University of Minnesota, Minneapolis, Minnesota, USA, 1986).

[103] Dyer, A. K. *A palynological investigation of the Late Quaternary vegetational history of the Baie Verte Peninsula*, *Northcentral Newfoundland*. (Memorial University of Newfoundland, 1986).

[104] Fall PL (1997). Timberline fluctuations and late Quaternary paleoclimates in the Southern Rocky Mountains, Colorado. Geol. Soc. Am. Bull.

[105] Nichols, H. *Palynological and paleoclimatic study of the late Quaternary displacements of the boreal forest-tundra ecotone in Keewatin and Mackenzie*, *N*.*W*.*T*., *Canada*. (University of Colorado, Boulder, 1975).

[106] Nichols JE (2014). Impacts of climate and vegetation change on carbon accumulation in a south-central Alaskan peatland assessed with novel organic geochemical techniques. The Holocene.

[107] Barnosky CW, Grimm EC, Wright HE (1987). Towards a postglacial history of the northern Great Plains: A review of the paleoecologic problems. Ann. Carnegie Mus..

[108] Rodrigues T, Grimalt JO, Abrantes F, Naughton F, Flores J-A (2010). The last glacial–interglacial transition (LGIT) in the western mid-latitudes of the North Atlantic: Abrupt sea surface temperature change and sea level implications. Quat. Sci. Rev..

[109] Huang X (2013). Paleotemperature variability in central China during the last 13 ka recorded by a novel microbial lipid proxy in the Dajiuhu peat deposit. The Holocene.

[110] Bjune A, Birks HJB, Seppä H (2004). Holocene vegetation and climate history on a continental-oceanic transect in northern Fennoscandia based on pollen and plant macrofossils. Boreas.

[111] Van Nieuwenhove N, Pearce C, Knudsen MF, Røy H, Seidenkrantz M-S (2018). Meltwater and seasonality influence on Subpolar Gyre circulation during the Holocene. Palaeogeogr. Palaeoclimatol. Palaeoecol.

[112] Polska-Jasiewicz Owa, M. & Latalowa, M. Palaeoecological events during the last 15000 years: regional syntheses of palaeoecological studies of lakes and mires in Europe. in *Palaeoecological events during the last 15000 years: regional syntheses of palaeoecological studies of lakes and mires in Europe* (eds. Berglund, B. E., Birks, H. J. B., Ralska-Jasiewicz Owa, M. & Wright, H. E.) 403–472 (J. Wiley and Sons, Chichester, 1996).

[113] Barnosky CW (1981). A record of Late Quaternary vegetation from Davis Lake, Southern Puget Lowland, Washington. Quat. Res.

[114] Szeicz JM, MacDonald GM (1991). Postglacial vegetation history of oak savanna in southern Ontario. Can. J. Bot..

[115] Richard, P. J. H., Larouche, A. C. & Bouchard, M. A. Age de la deglaciation finale et histoire postglaciaire de la vegetation dans la partie centrale du Nouveau-Quebec. *Geogr*. *Phys*. *Quat*. **36**, 63–90.

[116] Axford Y (2019). Holocene temperature history of northwest Greenland – With new ice cap constraints and chironomid assemblages from Deltasø. Quat. Sci. Rev..

[117] Porter TJ (2019). Recent summer warming in northwestern Canada exceeds the Holocene thermal maximum. Nat. Commun..

[118] Shuman BN, Marsicek J (2016). The structure of Holocene climate change in mid-latitude North America. Quat. Sci. Rev..

[119] Paterson WSB (1977). An oxygen-isotope climatic record from the Devon Island ice cap, arctic Canada. Nature.

[120] McAndrews JH (1984). Pollen analysis of the 1973 ice core from Devon Island Glacier, Canada. Quat. Res.

[121] Klemm J (2013). A pollen-climate transfer function from the tundra and taiga vegetation in Arctic Siberia and its applicability to a Holocene record. Palaeogeogr. Palaeoclimatol. Palaeoecol.

[122] Parrenin F (2007). 1-D-ice flow modelling at EPICA Dome C and Dome Fuji, East Antarctica. Clim. Past.

[123] Nilssen, E. J. *Klima-og vegetasjonshistoriske undersøkelser i Lofoten*. (University of Tromso, 1983).

[124] Winkler MG (1985). A 12,000-year history of vegetation and climate for Cape Cod, Massachusetts. Quat. Res.

[125] Seiwald A (1980). Beitraege zur Vegetationsgeschichte Tirols IV: Natzer Plateau - Villanderer Alm. Ber Nat-Med Ver. Innsbr.

[126] Crosta, X., Debret, M., Denis, D., Courty, M. A. & Ther, O. Holocene long- and short-term climate changes off Adélie Land, East Antarctica. *Geochem*. *Geophys*. *Geosystems* **8** (2007).

[127] Dahl-Jensen D (1998). Past temperatures directly from the Greenland Ice Sheet. Science.

[128] Dansgaard W (1982). A new Greenland deep ice core. Science.

[129] Rees ABH, Cwynar LC (2010). Evidence for early postglacial warming in Mount Field National Park, Tasmania. Quat. Sci. Rev..

[130] Stenni B (2010). The deuterium excess records of EPICA Dome C and Dronning Maud Land ice cores (East Antarctica). Quat. Sci. Rev..

[131] Langdon PG, Holmes N, Caseldine CJ (2008). Environmental controls on modern chironomid faunas from NW Iceland and implications for reconstructing climate change. J. Paleolimnol..

[132] Larocque-Tobler I, Heiri O, Wehrli M (2010). Late Glacial and Holocene temperature changes at Egelsee, Switzerland, reconstructed using subfossil chironomids. J. Paleolimnol..

[133] Gavin DG (2011). Abrupt Holocene climate change and potential response to solar forcing in western Canada. Quat. Sci. Rev..

[134] Schwamborn G, Meyer H, Fedorov G, Schirrmeister L, Hubberten H-W (2006). Ground ice and slope sediments archiving late Quaternary paleoenvironment and paleoclimate signals at the margins of El’gygytgyn Impact Crater, NE Siberia. Quat. Res.

[135] Flower BP, Hastings DW, Hill HW, Quinn TM (2004). Phasing of deglacial warming and Laurentide Ice Sheet meltwater in the Gulf of Mexico. Geology.

[136] Nichols, H. The post-glacial history of vegetation and climate at Ennadai Lake, Keewatin, and Lynn Lake, Manitoba (Canada). *Quat*. *Sci*. *J*. **181**, 176–197 (1967).

[137] Palmer MR (2003). A 23,000-year record of surface water pH and pCO_2_ in the western equatorial Pacific. Ocean. Science.

[138] Mackay AW (2012). Aquatic ecosystem responses to Holocene climate change and biome development in boreal, central Asia. Quat. Sci. Rev..

[139] Praetorius SK (2015). North Pacific deglacial hypoxic events linked to abrupt ocean warming. Nature.

[140] Foster LC (2016). Development of a regional glycerol dialkyl glycerol tetraether (GDGT)–temperature calibration for Antarctic and sub-Antarctic lakes. Earth Planet. Sci. Lett..

[141] Hu FS, Ito E, Brubaker LB, Anderson PM (1998). Ostracode geochemical record of Holocene climatic change and implications for vegetational response in the northwestern Alaska Range. Quat. Res.

[142] Linge H (2009). Stable isotope records for the last 10 000 years from Okshola cave (Fauske, northern Norway) and regional comparisons. Clim. Past.

[143] Albert, L.E. *Ferndale Bog and Natural Lake: Five Thousand Years of Environmental Change in Southeastern Oklahoma*. (Oklahoma Archeological Survey Studies in Oklahoma’s Past 7, 1981).

[144] Berglund BE (1966). Late-Quaternary vegetation in eastern Blekinge, south-eastern Sweden. Opera Bot.

[145] Lespez L (2008). Fluvial system evolution and environmental changes during the Holocene in the Mue valley (western France). Geomorphology.

[146] Rosenberg SM, Walker IR, Mathewes RW, Hallett DJ (2004). Midge-inferred Holocene climate history of two subalpine lakes in southern British Columbia, Canada. The Holocene.

[147] Webb, S. L. *The Holocene extension of the range of American Beech (Fagus grandifolia) into Wisconsin: Paleoecological evidence for long-distance seed dispersal*. (University of Minnesota, 1983).

[148] Samartin S (2017). Warm Mediterranean mid-Holocene summers inferred from fossil midge assemblages. Nat. Geosci..

[149] Setiawan RY (2015). The consequences of opening the Sunda Strait on the hydrography of the eastern tropical Indian Ocean. Paleoceanography.

[150] Mohtadi M, Steinke S, Lückge A, Groeneveld J, Hathorne EC (2010). Glacial to Holocene surface hydrography of the tropical eastern Indian Ocean. Earth Planet. Sci. Lett..

[151] Gibbons FT (2014). Deglacial δ18O and hydrologic variability in the tropical Pacific and Indian Oceans. Earth Planet. Sci. Lett..

[152] Kim J-H, Schneider RR, Müller PJ, Wefer G (2002). Interhemispheric comparison of deglacial sea-surface temperature patterns in Atlantic eastern boundary currents. Earth Planet. Sci. Lett..

[153] Kuhnert H (2014). Holocene tropical western Indian Ocean sea surface temperatures in covariation with climatic changes in the Indonesian region: Holocene Western Indian Ocean SSTs. Paleoceanography.

[154] Romahn S, Mackensen A, Groeneveld J, Pätzold J (2014). Deglacial intermediate water reorganization: New evidence from the Indian Ocean. Clim. Past.

[155] Kirst GJ, Schneider RR, Müller PJ, von Storch I, Wefer G (1999). Late Quaternary temperature variability in the Benguela Current system derived from alkenones. Quat. Res.

[156] Hollstein M (2018). Variations in Western Pacific Warm Pool surface and thermocline conditions over the past 110,000 years: Forcing mechanisms and implications for the glacial Walker circulation. Quat. Sci. Rev..

[157] Arz HW, Pätzold J, Wefer G (1998). Correlated millennial-scale changes in surface hydrography and terrigenous sediment yield inferred from Last-Glacial marine deposits off Northeastern Brazil. Quat. Res.

[158] Weldeab S, Schneider RR, Kölling M (2006). Deglacial sea surface temperature and salinity increase in the western tropical Atlantic in synchrony with high latitude climate instabilities. Earth Planet. Sci. Lett..

[159] Lamy F, Rühlemann C, Hebbeln D, Wefer G (2002). High- and low-latitude climate control on the position of the southern Peru-Chile Current during the Holocene. Paleoceanography.

[160] Arz HW, Gerhardt S, Pätzold J, Röhl U (2001). Millennial-scale changes of surface- and deep-water flow in the western tropical Atlantic linked to Northern Hemisphere high-latitude climate during the Holocene. Geology.

[161] Weldeab S, Schneider RR, Kölling M, Wefer G (2005). Holocene African droughts relate to eastern equatorial Atlantic cooling. Geology.

[162] Arz, H. W., Pätzold, J., Müller, P. J. & Moammar, M. O. Influence of Northern Hemisphere climate and global sea level rise on the restricted Red Sea marine environment during Termination I. *Paleoceanography* **18** (2003).

[163] Kim J-H (2004). North Pacific and North Atlantic sea-surface temperature variability during the Holocene. Quat. Sci. Rev..

[164] Schefuß E, Schouten S, Schneider RR (2005). Climatic controls on central African hydrology during the past 20,000 years. Nature.

[165] Weijers JWH, Schefuss E, Schouten S, Damste JSS (2007). Coupled thermal and hydrological evolution of tropical Africa over the last deglaciation. Science.

[166] Castañeda, I. S. *et al*. Millennial-scale sea surface temperature changes in the eastern Mediterranean (Nile River Delta region) over the last 27,000 years. *Paleoceanography* **25**, PA001470 (2010).

[167] Kim J-H (2012). Pronounced subsurface cooling of North Atlantic waters off Northwest Africa during Dansgaard–Oeschger interstadials. Earth Planet. Sci. Lett..

[168] Weldeab S, Lea DW, Oberhänsli H, Schneider RR (2014). Links between southwestern tropical Indian Ocean SST and precipitation over southeastern Africa over the last 17 kyr. Palaeogeogr. Palaeoclimatol. Palaeoecol.

[169] Schwab, C., Kinkel, H., Weinelt, M. & Repschläger, J. Coccolithophore paleoproductivity and ecology response to deglacial and Holocene changes in the Azores Current system. *Paleoceanography* **27** (2012).

[170] Farmer, J. R. *et al*. Western Arctic Ocean temperature variability during the last 8000 years. *Geophys*. *Res*. *Lett*. **38**, GL049714 (2011).

[171] Milecka, K. Pollen analysis of lake sediments in Giecz - The state of the investigation. in *Wstep do paleoecologii lednideiego parku**Krajobvazowego* (ed. Tobolski, K.) 147–150 (1991).

[172] Bortenschlager I (1976). Beitraege zur Vegetationsgeschichte Tirols II: Kufstein - Kitzbuehel - Pass Thurn. Ber Nat-Med Ver. Innsbr.

[173] Jung, S. J. A. *Wassermassenaustausch Zwischen Ne-Atlantik Und Nordmeer Während Der Letzten 300.000/80.000 Jahre Im Abbild Stabiler 0- und C-Isotope*. Berichte Aus Dem Sonderforschungsbereich Report No. 61 (Christian Albrechts University of Kiel, 1996).

[174] Kim J-H, Schneider RR, Hebbeln D, Müller PJ, Wefer G (2002). Last deglacial sea-surface temperature evolution in the Southeast Pacific compared to climate changes on the South American continent. Quat. Sci. Rev..

[175] Pelejero C, Grimalt JO, Heilig S, Kienast M, Wang L (1999). High-resolution U K37 temperature reconstructions in the South China Sea over the past 220 kyr. Paleoceanography.

[176] Kienast M (2001). Synchronous tropical South China Sea SST change and Greenland warming during deglaciation. Science.

[177] Schröder JF, Holbourn A, Kuhnt W, Küssner K (2016). Variations in sea surface hydrology in the southern Makassar Strait over the past 26 kyr. Quat. Sci. Rev..

[178] Sarnthein M (2008). Centennial-to-millennial-scale periodicities of Holocene climate and sediment injections off the western Barents shelf, 75°N. Boreas.

[179] Martrat B, Grimalt JO, Villanueva J, van Kreveld S, Sarnthein M (2003). Climatic dependence of the organic matter contributions in the north eastern Norwegian Sea over the last 15,000 years. Org. Geochem..

[180] Antonsson K, Brooks SJ, Seppä H, Telford RJ, Birks HJB (2006). Quantitative palaeotemperature records inferred from fossil pollen and chironomid assemblages from Lake Gilltjärnen, northern central Sweden. J. Quat. Sci.

[181] Kobashi T, Severinghaus JP, Kawamura K (2008). Argon and nitrogen isotopes of trapped air in the GISP2 ice core during the Holocene epoch (0–11,500 B.P.): Methodology and implications for gas loss processes. Geochim. Cosmochim. Acta.

[182] Cuffey KM, Clow GD (1997). Temperature, accumulation, and ice sheet elevation in central Greenland through the last deglacial transition. J. Geophys. Res. Oceans.

[183] Roesch M (2009). Zur vorgeschichtlichen Besiedlung und Landnutzung im noerdlichen Schwarzwald aufgrund vegetationsgeschichtlicher Untersuchungen in zwei Karseen. [Prehistoric settlement and land use history of the Northern Black Forest as indicated by pollenanalytical investigations in two cirque lakes]. Mitt Ver Forstl Standortskunde U Forstpflanzenzuechtung.

[184] Miotk, G. Badania palinologiczne osadow z polnocnego obrzeza jeziora Godziszewskiego kolo Tczewa/woj. *Gdanskie Fizjogr*. *Na Pol*. *Zach* **36**, 123–135 (1986).

[185] Anderson RS, Jacobson GL, Davis RB, Stuckenrath R (2008). Gould Pond, Maine: Late-glacial transitions from marine to upland environments. Boreas.

[186] Krisai R, Mayer W, Schroeck C, Tuerk R (2006). Das Gradenmoos in der Schobergruppe (NP Hohe Tauern, Kaerten) Vegetation und Entstehung. Carinth. II.

[187] Fuller JL (1997). Holocene forest dynamics in southern Ontario, Canada: Fine-resolution pollen data. Can. J. Bot..

[188] Visset, L. *Recherches Palynologiques sur la Vegetation Pleistocene et Holocene de Quelques Sites du District Phytogeographique de Basse-Loire*. (Bull. Soc. Sci. Nat. Ouest Fr., 1979).

[189] Salzer MW, Bunn AG, Graham NE, Hughes MK (2014). Five millennia of paleotemperature from tree-rings in the Great Basin, USA. Clim. Dyn.

[190] McCarthy, F. M. G. & McAndrews, J. H. Water levels in Lake Ontario 4230–2000 years B.P.: Evidence from Grenadier Pond, Toronto, Canada. *J*. *Paleolimnol*. **1**, 99–113 (1988).

[191] Evans, N. S. *An investigation of the Holocene pollen record from the Grey Islands*, *Newfoundland*. (Memorial University of Newfoundland, 2002).

[192] Johnsen SJ, Dahl-Jensen D, Dansgaard W, Gundestrup N (1995). Greenland palaeotemperatures derived from GRIP bore hole temperature and ice core isotope profiles. Tellus B Chem. Phys. Meteorol.

[193] Allen JRM, Long AJ, Ottley CJ, Graham Pearson D, Huntley B (2007). Holocene climate variability in northernmost Europe. Quat. Sci. Rev..

[194] Self AE, Jones VJ, Brooks SJ (2015). Late Holocene environmental change in arctic western Siberia. The Holocene.

[195] Samson, C. R., Sikes, E. L. & Howard, W. R. Deglacial paleoceanographic history of the Bay of Plenty, New Zealand. *Paleoceanography* **20**, PA001088 (2005).

[196] Heeb K, Welten M (1972). Moore und Vegetationsgeschichte der Schwarzenegg und des Molassevorlandes zwischen dem Aaretal unterhalb Thun und dem obern Emmental. Mitteilungen der Naturforschenden Gesellschaft in Bern. N. F.

[197] McKay NP, Kaufman DS (2009). Holocene climate and glacier variability at Hallet and Greyling Lakes, Chugach Mountains, south-central Alaska. J. Paleolimnol..

[198] Caseldine C, Langdon P, Holmes N (2006). Early Holocene climate variability and the timing and extent of the Holocene thermal maximum (HTM) in northern Iceland. Quat. Sci. Rev..

[199] Cwynar LC (1982). A Late-Quaternary vegetation history from Hanging Lake, Northern Yukon. Ecol. Monogr.

[200] Zheng Y (2017). Atmospheric connections with the North Atlantic enhanced the deglacial warming in northeast China. Geology.

[201] Geirsdóttir Á, Miller GH, Larsen DJ, Ólafsdóttir S (2013). Abrupt Holocene climate transitions in the northern North Atlantic region recorded by synchronized lacustrine records in Iceland. Quat. Sci. Rev..

[202] Coutard, S. & Clet-Pellerin, M. Evolution de la sedimentation et de la vegetation pendant l’Holocene dans les marais arriere-littoraux du Val de Saire (Cotentin, Normandie). in *L’erosion entre Societe*, *Climat et Paleoenvironnements* 271–278 (Presses Universitaires Blaise Pascal, Clermont-Ferrand, coll., Nature-Societe, 2006).

[203] Brown Macpherson J (1982). Postglacial vegetational history of the eastern Avalon Peninsula, Newfoundland, and Holocene climatic change along the eastern Canadian seaboard. Géographie Phys. Quat.

[204] Chang J, Zhang E, Liu E, Shulmeister J (2017). Summer temperature variability inferred from subfossil chironomid assemblages from the south-east margin of the Qinghai–Tibetan Plateau for the last 5000 years. The Holocene.

[205] Wang C (2018). Holocene temperature and hydrological changes reconstructed by bacterial 3-hydroxy fatty acids in a stalagmite from central China. Quat. Sci. Rev..

[206] Potito AP, Porinchu DF, MacDonald GM, Moser KA (2006). A late Quaternary chironomid-inferred temperature record from the Sierra Nevada, California, with connections to northeast Pacific sea surface temperatures. Quat. Res.

[207] Heiri O, Ilyashuk B, Millet L, Samartin S, Lotter AF (2015). Stacking of discontinuous regional palaeoclimate records: Chironomid-based summer temperatures from the Alpine region. The Holocene.

[208] Luoto TP, Kultti S, Nevalainen L, Sarmaja-Korjonen K (2010). Temperature and effective moisture variability in southern Finland during the Holocene quantified with midge-based calibration models. J. Quat. Sci.

[209] Schmidt S (2011). Chironomids as indicators of the Holocene climatic and environmental history of two lakes in Northeast Greenland: Chironomids as indicators of the Holocene climatic and environmental history, NE-Greenland. Boreas.

[210] Wagner B (2008). A multidisciplinary study of Holocene sediment records from Hjort Sø on Store Koldewey, Northeast Greenland. J. Paleolimnol..

[211] Mohtadi M (2008). Deglacial pattern of circulation and marine productivity in the upwelling region off central-south Chile. Earth Planet. Sci. Lett..

[212] de Vernal A (2013). Dinocyst-based reconstructions of sea ice cover concentration during the Holocene in the Arctic Ocean, the northern North Atlantic Ocean and its adjacent seas. Quat. Sci. Rev..

[213] Solignac S (2008). Reorganization of the upper ocean circulation in the mid-Holocene in the northeastern Atlantic. Can. J. Earth Sci..

[214] Giesecke T (2008). Exploring Holocene continentality changes in Fennoscandia using present and past tree distributions. Quat. Sci. Rev..

[215] Litt T, Schölzel C, Kühl N, Brauer A (2009). Vegetation and climate history in the Westeifel Volcanic Field (Germany) during the past 11 000 years based on annually laminated lacustrine maar sediments. Boreas.

[216] McGlone MS, Turney CSM, Wilmshurst JM, Renwick J, Pahnke K (2010). Divergent trends in land and ocean temperature in the Southern Ocean over the past 18,000 years. Nat. Geosci..

[217] Cwynar LC, Spear RW (1991). Reversion of forest to tundra in the central Yukon. Ecology.

[218] Keigwin LD, Jones GA (1995). The marine record of deglaciation from the continental margin off Nova Scotia. Paleoceanography.

[219] Andrews JT, Keigwin L, Hall F, Jennings AE (1999). Abrupt deglaciation events and Holocene palaeoceanography from high-resolution cores, Cartwright Saddle, Labrador Shelf, Canada. J. Quat. Sci.

[220] Hillaire-Marcel C, Vernal A, de, Bilodeau G, Wu G (1994). Isotope stratigraphy, sedimentation rates, deep circulation, and carbonate events in the Labrador Sea during the last ~ 200 ka. Can. J. Earth Sci..

[221] Clegg BF, Kelly R, Clarke GH, Walker IR, Hu FS (2011). Nonlinear response of summer temperature to Holocene insolation forcing in Alaska. Proc. Natl. Acad. Sci..

[222] Wen R (2010). Holocene precipitation and temperature variations in the East Asian monsoonal margin from pollen data from Hulun Lake in northeastern Inner Mongolia, China. Boreas.

[223] Weninger JM, McAndrews JH (1989). Late Holocene aggradation in the lower Humber River valley, Toronto, Ontario. Can. J. Earth Sci..

[224] Zhao C (2013). Holocene temperature fluctuations in the northern Tibetan Plateau. Quat. Res.

[225] Hájková P (2016). A first chironomid-based summer temperature reconstruction (13–5 ka BP) around 49°N in inland Europe compared with local lake development. Quat. Sci. Rev..

[226] Massa C (2012). A multiproxy evaluation of Holocene environmental change from Lake Igaliku, South Greenland. J. Paleolimnol..

[227] Björck S (1985). Deglaciation chronology and revegetation in northwestern Ontario. Can. J. Earth Sci..

[228] Kerwin MW, Overpeck JT, Webb RS, Anderson KH (2004). Pollen-based summer temperature reconstructions for the eastern Canadian boreal forest, subarctic, and Arctic. Quat. Sci. Rev..

[229] Emeis K-C, Struck U, Blanz T, Kohly A, Voβ M (2003). Salinity changes in the central Baltic Sea (NW Europe) over the last 10000 years. The Holocene.

[230] Alwin, B. C. *Vegetation history of the Sugar Hills area*, *Itasca Co*., *Minnesota*. (University of Minnesota, 1982).

[231] Asplund H, Vuorela I (1989). Settlement studies in Kemioe - archaeological problems and palynological evidence. Fennoskandia Archaeol.

[232] Miller GH, Wolfe AP, Briner JP, Sauer PE, Nesje A (2005). Holocene glaciation and climate evolution of Baffin Island, Arctic Canada. Quat. Sci. Rev..

[233] Mulvaney R (2012). Recent Antarctic Peninsula warming relative to Holocene climate and ice-shelf history. Nature.

[234] Yu S-Y (2013). Quantitative reconstruction of mid- to late-Holocene climate in NE China from peat cellulose stable oxygen and carbon isotope records and mechanistic models. The Holocene.

[235] Hald M (2007). Variations in temperature and extent of Atlantic Water in the northern North Atlantic during the Holocene. Quat. Sci. Rev..

[236] Solignac, S., Giraudeau, J. & de Vernal, A. Holocene sea surface conditions in the western North Atlantic: Spatial and temporal heterogeneities. *Paleoceanography* **21**, PA001175 (2006).

[237] Fortin M-C, Gajewski K (2016). Multiproxy paleoecological evidence of Holocene climatic changes on the Boothia Peninsula, Canadian Arctic. Quat. Res.

[238] Zabenskie S, Gajewski K (2007). Post-Glacial climatic change on Boothia Peninsula, Nunavut, Canada. Quat. Res.

[239] Roberts J (2016). Evolution of South Atlantic density and chemical stratification across the last deglaciation. Proc. Natl. Acad. Sci..

[240] Bendle JAP, Rosell-Melé A (2007). High-resolution alkenone sea surface temperature variability on the north Icelandic Shelf: Implications for Nordic Seas palaeoclimatic development during the Holocene. The Holocene.

[241] Fallu M-A, Pienitz R, Walker IR, Lavoie M (2005). Paleolimnology of a shrub-tundra lake and response of aquatic and terrestrial indicators to climatic change in arctic Québec, Canada. Palaeogeogr. Palaeoclimatol. Palaeoecol.

[242] Rankama T, Vuorela I (1988). Between inland and coast in Metal Age Finland - human impact on the primeval forests of Southern Häme during the Iron Age. Memo. Soc. Fauna Flora Fenn..

[243] Martín-Chivelet J, Muñoz-García MB, Edwards RL, Turrero MJ, Ortega AI (2011). Land surface temperature changes in Northern Iberia since 4000yrBP, based on δ13C of speleothems. Glob. Planet. Change.

[244] Kremenetski CV (1995). Holocene vegetation and climate history of southwestern Ukraine. Rev. Palaeobot. Palynol..

[245] Porter, S. C. & Sauchyn, M. A. *Moose Mountain Palynology Study: Final Report*. Unpublished. (1992).

[246] Fall, P. L. Holocene dynamics of the subalpine forest in central Colorado. *Am*. *Assoc*. *Stratigr*. *Palynol*. *Contrib*. *Ser*. **16**, 31–46 (1985).

[247] Jones VJ (2011). The influence of Holocene tree-line advance and retreat on an arctic lake ecosystem: A multi-proxy study from Kharinei Lake, northeastern European Russia. J. Paleolimnol..

[248] Väliranta M (2015). Plant macrofossil evidence for an early onset of the Holocene summer thermal maximum in northernmost Europe. Nat. Commun..

[249] Syrykh LS, Nazarova LB, Herzschuh U, Subetto DA, Grekov IM (2017). Reconstruction of palaeoecological and palaeoclimatic conditions of the Holocene in the south of the Taimyr according to an analysis of lake sediments. Contemp. Probl. Ecol.

[250] Baker JL, Lachniet MS, Chervyatsova O, Asmerom Y, Polyak VJ (2017). Holocene warming in western continental Eurasia driven by glacial retreat and greenhouse forcing. Nat. Geosci..

[251] Carlson AE (2008). Subtropical Atlantic salinity variability and Atlantic meridional circulation during the last deglaciation. Geology.

[252] Antonarakou, A. *et al*. Biotic and geochemical (δ^18^O, δ^13^C, Mg/Ca, Ba/Ca) responses of *Globigerinoides ruber* morphotypes to upper water column variations during the last deglaciation, Gulf of Mexico. *Geochim. Cosmochim. Acta* **170**, 69–93 (2015).

[253] Elmore AC, Wright JD, Southon J (2015). Continued meltwater influence on North Atlantic Deep Water instabilities during the early Holocene. Mar. Geol.

[254] Schmidt, M. W. & Lynch‐Stieglitz, J. Florida Straits deglacial temperature and salinity change: Implications for tropical hydrologic cycle variability during the Younger Dryas. *Paleoceanography* **26**, PA002157 (2011).

[255] Schmidt, M. W., Weinlein, W. A., Marcantonio, F. & Lynch-Stieglitz, J. Solar forcing of Florida Straits surface salinity during the early Holocene. *Paleoceanography* **27**, PA002284 (2012).

[256] Bova SC (2015). Links between eastern equatorial Pacific stratification and atmospheric CO_2_ rise during the last deglaciation. Paleoceanography.

[257] Ciais P (1992). Evidence for an early Holocene climatic optimum in the Antarctic deep ice-core record. Clim. Dyn.

[258] Herzschuh U, Kramer A, Mischke S, Zhang C (2009). Quantitative climate and vegetation trends since the late glacial on the northeastern Tibetan Plateau deduced from Koucha Lake pollen spectra. Quat. Res.

[259] Fortin M-C, Gajewski K (2010). Postglacial environmental history of western Victoria Island, Canadian Arctic. Quat. Sci. Rev..

[260] Peros MC, Gajewski K (2008). Holocene climate and vegetation change on Victoria Island, western Canadian Arctic. Quat. Sci. Rev..

[261] Kawahata H (2009). Changes of environments and human activity at the Sannai-Maruyama ruins in Japan during the mid-Holocene Hypsithermal climatic interval. Quat. Sci. Rev..

[262] Boldt BR, Kaufman DS, McKay NP, Briner JP (2015). Holocene summer temperature reconstruction from sedimentary chlorophyll content, with treatment of age uncertainties, Kurupa Lake, Arctic Alaska. The Holocene.

[263] Chakraborty K, Finkelstein SA, Desloges JR, Chow NA (2010). Holocene paleoenvironmental changes inferred from diatom assemblages in sediments of Kusawa Lake, Yukon Territory, Canada. Quat. Res.

[264] Kubota, Y. *et al*. Variations of East Asian summer monsoon since the last deglaciation based on Mg/Ca and oxygen isotope of planktic foraminifera in the northern East China Sea. *Paleoceanography* **25**, PA001891 (2010).

[265] Biskaborn BK (2016). Late Quaternary vegetation and lake system dynamics in north-eastern Siberia: Implications for seasonal climate variability. Quat. Sci. Rev..

[266] Hardy W (2018). Quantification of last glacial-Holocene net primary productivity and upwelling activity in the equatorial eastern Atlantic with a revised modern dinocyst database. Palaeogeogr. Palaeoclimatol. Palaeoecol.

[267] Bajolle L (2018). Major postglacial summer temperature changes in the central coniferous boreal forest of Quebec (Canada) inferred using chironomid assemblages. J. Quat. Sci.

[268] Hausmann S (2011). Diatom-inferred wind activity at Lac du Sommet, southern Québec, Canada: A multiproxy paleoclimate reconstruction based on diatoms, chironomids and pollen for the past 9500 years. The Holocene.

[269] Richard, P. J. H. *Paleophytogeographie postglaciaire en Ungava par l’analyse pollinique*. in 154 (1981).

[270] Labelle C, Richard PJH (1984). Histoire postglaciaire de la végétation dans la région de Mont-Saint-Pierre, Gaspésie, Québec. Géographie Phys. Quat.

[271] MacDonald GM (1987). Postglacial vegetation history of the Mackenzie River Basin. Quat. Res.

[272] Samson, G. *Prehistorie du Mushuau Nipi*, *Nouveau-Québec: Étude du mode d’adaptation à l’intérieur des terres hémi-arctiques*. (University of Toronto, 1983).

[273] Shane LCK, Anderson KH (1993). Intensity, gradients and reversals in late glacial environmental change in east-central north America. Quat. Sci. Rev..

[274] Allen JRM, Huntley B, Watts WA (1996). The vegetation and climate of northwest Iberia over the last 14,000 years. J. Quat. Sci.

[275] Finsinger W (2010). Early to mid-Holocene climate change at Lago dell’Accesa (central Italy): Climate signal or anthropogenic bias?. J. Quat. Sci.

[276] Markgraf V, Webb RS, Anderson KH, Anderson L (2002). Modern pollen/climate calibration for southern South America. Palaeogeogr. Palaeoclimatol. Palaeoecol.

[277] Punyasena SW, Mayle FE, McElwain JC (2008). Quantitative estimates of glacial and Holocene temperature and precipitation change in lowland Amazonian Bolivia. Geology.

[278] Eisner WR, Törnqvist TE, Koster EA, Bennike O, van Leeuwen JFN (1995). Paleoecological studies of a Holocene lacustrine record from the Kangerlussuaq (Søndre Strømfjord) region of west Greenland. Quat. Res.

[279] Shemesh A (2001). Holocene climatic change in Swedish Lapland inferred from an oxygen-isotope record of lacustrine biogenic silica. The Holocene.

[280] Gajewski K (1991). Représentation pollinique actuelle à la limite des arbres au Nouveau-Québec. Can. J. Earth Sci..

[281] Zhilich S, Rudaya N, Krivonogov S, Nazarova L, Pozdnyakov D (2017). Environmental dynamics of the Baraba forest-steppe (Siberia) over the last 8000 years and their impact on the types of economic life of the population. Quat. Sci. Rev..

[282] Garralla S, Gajewski K (1992). Holocene vegetation history of the boreal forest near Chibougamau, central Quebec. Can. J. Bot..

[283] Gajewski K, Payette S, Ritchie JC (1993). Holocene Vegetation History at the Boreal-Forest-Shrub-Tundra Transition in North-Western Quebec. J. Ecol..

[284] Engstrom DR, Hansen BCS (1985). Postglacial vegetational change and soil development in southeastern Labrador as inferred from pollen and chemical stratigraphy. Can. J. Bot..

[285] Woltering M (2014). Glacial and Holocene terrestrial temperature variability in subtropical east Australia as inferred from branched GDGT distributions in a sediment core from Lake McKenzie. Quat. Res.

[286] Johnson TC (2016). A progressively wetter climate in southern East Africa over the past 1.3 million years. Nature.

[287] Opitz S, Zhang C, Herzschuh U, Mischke S (2015). Climate variability on the south-eastern Tibetan Plateau since the Lateglacial based on a multiproxy approach from Lake Naleng – comparing pollen and non-pollen signals. Quat. Sci. Rev..

[288] Palmer S, Walker I, Heinrichs M, Hebda R, Scudder G (2002). Postglacial midge community change and Holocene palaeotemperature reconstructions near treeline, southern British Columbia (Canada). J. Paleolimnol.

[289] Hou J (2016). Large Holocene summer temperature oscillations and impact on the peopling of the northeastern Tibetan Plateau. Geophys. Res. Lett..

[290] Futyma RP, Miller NG (1986). Stratigraphy and genesis of the Lake Sixteen peatland, northern Michigan. Can. J. Bot..

[291] Porinchu DF (2019). Evidence of abrupt climate change at 9.3 ka and 8.2 ka in the central Canadian Arctic: Connection to the North Atlantic and Atlantic Meridional Overturning Circulation. Quat. Sci. Rev..

[292] Berke MA (2012). Molecular records of climate variability and vegetation response since the Late Pleistocene in the Lake Victoria basin, East Africa. Quat. Sci. Rev..

[293] Van Zant K (1979). Late Glacial and postglacial pollen and plant macrofossils from Lake West Okoboji, northwestern Iowa. Quat. Res.

[294] Herzschuh U, Borkowski J, Schewe J, Mischke S, Tian F (2014). Moisture-advection feedback supports strong early-to-mid Holocene monsoon climate on the eastern Tibetan Plateau as inferred from a pollen-based reconstruction. Palaeogeogr. Palaeoclimatol. Palaeoecol.

[295] Pivel MAG, Santarosa ACA, Toledo FAL, Costa KB (2013). The Holocene onset in the southwestern South. Atlantic. Palaeogeogr. Palaeoclimatol. Palaeoecol.

[296] Helama S (2009). Summer temperature variations in Lapland during the Medieval Warm Period and the Little Ice Age relative to natural instability of thermohaline circulation on multi-decadal and multi-centennial scales. J. Quat. Sci.

[297] Dahl-Jensen D, Morgan VI, Elcheikh A (1999). Monte Carlo inverse modelling of the Law Dome (Antarctica) temperature profile. Ann. Glaciol..

[298] Barbier, D. Histoire de la vegetation du nord-mayennais de la fin du Weichselien a l’aube du XXIeme siecle. Mise en evidence d’un Tardiglaciaire armoricain. *Interactions Homme-Milieu*. (Universite de Nantes, 1999).

[299] Clerc, J. *Recherches pollenanalytiques sur la paleo-ecologie Tardiglaciaire et Holocene du Bas-Dauphine*. (Universite St. Jerome, 1988).

[300] Makohonienko, M. & Walanus, A. Analizy numeryczne wyników badán palinologicznych osadów Jeziora Lednickiego [Numerical analyses of the pollen analytical research results of the sediments from Lednica Lake] in *Wstep do paleoekologii Lednideiego Parku Krajobvazowego* (ed. Tobolski, K.), 63–70 (University Press, Poznan, Poland, 1991).

[301] Litt, T. & Tobolski, K. Beitraege zur postglazialen Vegetaionsgeschichte im Lednica-Gebiet. in *Wstep do paleoekologii Lednickiego Parku Krajobrazowego* (ed. Tobolski, K.) 57–61 (University Press, Poznan, Poland, 1991).

[302] Lachniet MS, Denniston RF, Asmerom Y, Polyak VJ (2014). Orbital control of western North America atmospheric circulation and climate over two glacial cycles. Nat. Commun..

[303] Solovieva N (2015). The Holocene environmental history of a small coastal lake on the north-eastern Kamchatka Peninsula. Glob. Planet. Change.

[304] Koff, T. *Reconstruction of Palaeogeographical Conditions in NE Estonia on the Basis of Bog and Lake Deposits*. (Estonia-Finnish seminar on environmental questions, 99–115, 1989).

[305] Cwynar LC (1990). A late Quaternary vegetation history from Lily Lake, Chilkat Peninsula, southeast Alaska. Can. J. Bot..

[306] Billard C, Clet-Pellerin M, Lautridou J-P, Giffault M (1995). Un site protohistorique littoral dans le havre de la Vanlée à Lingreville et Bricqueville-sur-Mer (Manche). Rev. Archéologique Ouest.

[307] Moser KA, MacDonald GM (1990). Holocene vegetation change at treeline north of Yellowknife, northwest Territories, Canada. Quat. Res.

[308] Birks HJB, Madsen BJ (1979). Flandrian vegetational history of Little Loch Roag, Isle of Lewis, Scotland. J. Ecol..

[309] Berner, K. S., Koç, N., Divine, D., Godtliebsen, F. & Moros, M. A decadal-scale Holocene sea surface temperature record from the subpolar North Atlantic constructed using diatoms and statistics and its relation to other climate parameters. *Paleoceanography* **23**, PA001339 (2008).

[310] Ammann B (1985). Introduction and palynology: Vegetational history and core correlation at Lobsigensee (Swiss Plateau). Diss. Bot.

[311] Dalton C (2005). A multi-proxy study of lake-development in response to catchment changes during the Holocene at Lochnagar, north-east Scotland. Palaeogeogr. Palaeoclimatol. Palaeoecol.

[312] MacDonald GM, Cwynar LC (1985). A fossil pollen based reconstruction of the late Quaternary history of lodgepole pine (*Pinus contorta* ssp. *latifolia*) in the western interior of Canada. Can. J. For. Res.

[313] Kaufman DS (2012). A multi-proxy record of the Last Glacial Maximum and last 14,500 years of paleoenvironmental change at Lone Spruce Pond, southwestern Alaska. J. Paleolimnol..

[314] Jacobson, G. L., Davis, R. B., Anderson, R. S., Tolonen, M. & Stuckenrath, R., Jr. Post-glacial vegetation of the coastal lowlands of Maine. Unpublished manuscript. (n.d.).

[315] Taylor KJ, McGinley S, Potito AP, Molloy K, Beilman DW (2018). A mid to late Holocene chironomid-inferred temperature record from northwest Ireland. Palaeogeogr. Palaeoclimatol. Palaeoecol.

[316] Pellatt MG, Mathewes RW (1994). Paleoecology of postglacial tree line fluctuations on the Queen Charlotte Islands, Canada. Écoscience.

[317] Kvavadze EV, Efremov YV, Bukreeva GV, Akatov VV (1994). Palynological characteristic of the series of lacustrine and paludal deposites of the Holocene in the headwaters of the Zakan river (Western Caucsus). Bull. Georgian Acad. Sci.

[318] Riethdorf J-R, Max L, Nürnberg D, Lembke-Jene L, Tiedemann R (2013). Deglacial development of (sub) sea surface temperature and salinity in the subarctic northwest Pacific: Implications for upper-ocean stratification. Paleoceanography.

[319] Andreev AA (2005). Holocene environmental history recorded in Lake Lyadhej-To sediments, Polar Urals, Russia. Palaeogeogr. Palaeoclimatol. Palaeoecol.

[320] Nichols H (1967). Central Canadian palynology and its relevance to northwestern Europe in the late quaternary period. Rev. Palaeobot. Palynol..

[321] Ritchie JC (1977). The Modern and Late Quaternary vegetation of the Campbell-Dolomite Uplands, near Inuvik, N.W.T., Canada. Ecol. Monogr.

[322] Salvatteci R, Schneider RR, Blanz T, Mollier‐Vogel E (2019). Deglacial to Holocene ocean temperatures in the Humboldt Current System as indicated by alkenone paleothermometry. Geophys. Res. Lett..

[323] Rühlemann C, Mulitza S, Müller PJ, Wefer G, Zahn R (1999). Warming of the tropical Atlantic Ocean and slowdown of thermohaline circulation during the last deglaciation. Nature.

[324] Eynaud, F. *et al*. Position of the Polar Front along the western Iberian margin during key cold episodes of the last 45 ka. *Geochem*. *Geophys*. *Geosystems* **10** (2009).

[325] Emeis K-C, Dawson AG (2003). Holocene palaeoclimate records over Europe and the North Atlantic. The Holocene.

[326] Wang YV (2013). Northern and southern hemisphere controls on seasonal sea surface temperatures in the Indian Ocean during the last deglaciation. Paleoceanography.

[327] Nürnberg D (2015). Sea surface and subsurface circulation dynamics off equatorial Peru during the last 17 kyr. Paleoceanography.

[328] Tonkov S, Bozilova EDB (1992). Pollen analysis of peat bog in Maleshevska mountain (SW Bulgaria). Annu. Sofia Univ. Fac. Biol.

[329] Dinel H, Richard PJH, Levésque PEM, Larouche A (1986). Origine et évolution du marais tourbeux de Keswick, Ontario, par l’analyse pollinique et macrofossile. Can. J. Earth Sci..

[330] Xu J, Holbourn A, Kuhnt W, Jian Z, Kawamura H (2008). Changes in the thermocline structure of the Indonesian outflow during Terminations I and II. Earth Planet. Sci. Lett..

[331] Steinke S (2008). Proxy dependence of the temporal pattern of deglacial warming in the tropical South China Sea: Toward resolving seasonality. Quat. Sci. Rev..

[332] Harada N (2006). Rapid fluctuation of alkenone temperature in the southwestern Okhotsk Sea during the past 120 ky. Glob. Planet. Change.

[333] Isono D (2009). The 1500-year climate oscillation in the midlatitude North Pacific during the Holocene. Geology.

[334] Sarnthein M (2004). Mid Holocene origin of the sea-surface salinity low in the subarctic North Pacific. Quat. Sci. Rev..

[335] Martrat B (2007). Four climate cycles of recurring deep and surface water destabilizations on the Iberian Margin. Science.

[336] Salgueiro E (2014). Past circulation along the western Iberian margin: A time slice vision from the Last Glacial to the Holocene. Quat. Sci. Rev..

[337] Hill TM (2006). Pre-Bølling warming in Santa Barbara Basin, California: surface and intermediate water records of early deglacial warmth. Quat. Sci. Rev..

[338] McClymont, E. L. *et al*. Sea-surface temperature records of Termination 1 in the Gulf of California: Challenges for seasonal and interannual analogues of tropical Pacific climate change. *Paleoceanography* **27**, PA002226 (2012).

[339] Ziegler M, Nürnberg D, Karas C, Tiedemann R, Lourens LJ (2008). Persistent summer expansion of the Atlantic Warm Pool during glacial abrupt cold events. Nat. Geosci..

[340] Dyez KA, Zahn R, Hall IR (2014). Multicentennial Agulhas leakage variability and links to North Atlantic climate during the past 80,000 years: Agulhas links to Atlantic climate. Paleoceanography.

[341] Lopes dos Santos RA (2013). Comparison of organic (U K’ 37, TEX H 86, LDI) and faunal proxies (foraminiferal assemblages) for reconstruction of late Quaternary sea surface temperature variability from offshore southeastern Australia. Paleoceanography.

[342] Calvo, E., Pelejero, C., De Deckker, P. & Logan, G. A. Antarctic deglacial pattern in a 30 kyr record of sea surface temperature offshore South Australia. *Geophys*. *Res*. *Lett*. **34**, GL029937 (2007).

[343] Weldeab S, Lea DW, Schneider RR, Andersen N (2007). 155,000 years of West African Monsoon and ocean thermal evolution. Science.

[344] Kim, J.-H. *et al*. Holocene subsurface temperature variability in the eastern Antarctic continental margin. *Geophys*. *Res*. *Lett*. **39**, GL051157 (2012).

[345] Bolliet, T. *et al*. Mindanao Dome variability over the last 160 kyr: Episodic glacial cooling of the West Pacific Warm Pool. *Paleoceanography* **26**, PA001966 (2011).

[346] Fraser N (2014). Precipitation variability within the West Pacific Warm Pool over the past 120 ka: Evidence from the Davao Gulf, southern Philippines. Paleoceanography.

[347] Gottschalk J, Skinner LC, Waelbroeck C (2015). Contribution of seasonal sub-Antarctic surface water variability to millennial-scale changes in atmospheric CO2 over the last deglaciation and Marine Isotope Stage 3. Earth Planet. Sci. Lett..

[348] Montade V, Peyron O, Favier C, Francois JP, Haberle SG (2019). A pollen-climate calibration from western Patagonia for palaeoclimatic reconstructions. J. Quat. Sci.

[349] Bard E, Rostek F, Sonzogni C (1997). Interhemispheric synchrony of the last deglaciation inferred from alkenone palaeothermometry. Nature.

[350] Levi, C. *et al*. Low-latitude hydrological cycle and rapid climate changes during the last deglaciation. *Geochem*. *Geophys*. *Geosystems* **8**, GC001514 (2007).

[351] Labracherie M (1989). The last deglaciation in the Southern Ocean. Paleoceanography.

[352] Sarnthein M, Winn K, Duplessy J-C, Fontugne MR (1988). Global variations of surface ocean productivity in low and mid latitudes: Influence on CO_2_ reservoirs of the deep ocean and atmosphere during the last 21,000 years. Paleoceanography.

[353] Sicre M (2005). Mid-latitude Southern Indian Ocean response to Northern Hemisphere Heinrich events. Earth Planet. Sci. Lett..

[354] Eynaud, F. *et al*. New constraints on European glacial freshwater releases to the North Atlantic Ocean. *Geophys*. *Res*. *Lett*. **39**, GL052100 (2012).

[355] Risebrobakken, B., Jansen, E., Andersson, C., Mjelde, E. & Hevrøy, K. A high-resolution study of Holocene paleoclimatic and paleoceanographic changes in the Nordic Seas. *Paleoceanography* **18** (2003).

[356] Marchal O (2002). Apparent long-term cooling of the sea surface in the northeast Atlantic and Mediterranean during the Holocene. Quat. Sci. Rev..

[357] Cacho I (1999). Dansgaard-Oeschger and Heinrich event imprints in Alboran Sea paleotemperatures. Paleoceanography.

[358] Pahnke K (2003). 340,000-Year centennial-scale marine record of Southern Hemisphere Climatic Oscillation. Science.

[359] Pahnke, K. & Sachs, J. P. Sea surface temperatures of southern midlatitudes 0-160 kyr B.P. *Paleoceanography* **21**, PA001191 (2006).

[360] Rosenthal, Y., Oppo, D. W. & Linsley, B. K. The amplitude and phasing of climate change during the last deglaciation in the Sulu Sea, western equatorial Pacific. *Geophys*. *Res*. *Lett*. **30**, GL016612 (2003).

[361] Shintani T, Yamamoto M, Chen M-T (2011). Paleoenvironmental changes in the northern South China Sea over the past 28,000 years: A study of TEX86-derived sea surface temperatures and terrestrial biomarkers. J. Asian Earth Sci..

[362] Yamamoto M, Sai H, Chen M-T, Zhao M (2013). The East Asian winter monsoon variability in response to precession during the past 150 000 yr. Clim. Past.

[363] Fan W (2018). Variability of the Indonesian throughflow in the Makassar Strait over the last 30 ka. Sci. Rep.

[364] Stott L, Timmermann A, Thunell R (2007). Southern Hemisphere and deep-sea warming led deglacial atmospheric CO2 rise and tropical warming. Science.

[365] Dang, H., Jian, Z., Bassinot, F., Qiao, P. & Cheng, X. Decoupled Holocene variability in surface and thermocline water temperatures of the Indo-Pacific Warm Pool. *Geophys*. *Res*. *Lett*. **39**, GL050154 (2012).

[366] Ijiri A (2005). Paleoenvironmental changes in the northern area of the East China Sea during the past 42,000 years. Palaeogeogr. Palaeoclimatol. Palaeoecol.

[367] Farmer, E. J., Chapman, M. R. & Andrews, J. E. Centennial-scale Holocene North Atlantic surface temperatures from Mg/Ca ratios in *Globigerina bulloides*. *Geochem*. *Geophys*. *Geosystems* **9**, GC002199 (2008).

[368] Kristjánsdóttir GB, Moros M, Andrews JT, Jennings AE (2017). Holocene Mg/Ca, alkenones, and light stable isotope measurements on the outer North Iceland shelf (MD99-2269): A comparison with other multi-proxy data and sub-division of the Holocene. The Holocene.

[369] Jennings A, Andrews J, Pearce C, Wilson L, Ólfasdótttir S (2015). Detrital carbonate peaks on the Labrador shelf, a 13–7ka template for freshwater forcing from the Hudson Strait outlet of the Laurentide Ice Sheet into the subpolar gyre. Quat. Sci. Rev..

[370] Moossen H, Bendle J, Seki O, Quillmann U, Kawamura K (2015). North Atlantic Holocene climate evolution recorded by high-resolution terrestrial and marine biomarker records. Quat. Sci. Rev..

[371] Justwan A, Koç N, Jennings AE (2008). Evolution of the Irminger and East Icelandic Current systems through the Holocene, revealed by diatom-based sea surface temperature reconstructions. Quat. Sci. Rev..

[372] Eldevik T (2014). A brief history of climate – the northern seas from the Last Glacial Maximum to global warming. Quat. Sci. Rev..

[373] Jennings A, Andrews J, Wilson L (2011). Holocene environmental evolution of the SE Greenland Shelf north and south of the Denmark Strait: Irminger and East Greenland current interactions. Quat. Sci. Rev..

[374] Benway, H. M., Mix, A. C., Haley, B. A. & Klinkhammer, G. P. Eastern Pacific Warm Pool paleosalinity and climate variability: 0-30 kyr. *Paleoceanography* **21**, PA001208 (2006).

[375] Nazarova LB, Subetto DA, Syrykh LS, Grekov IM, Leontev PA (2018). Reconstructions of paleoecological and paleoclimatic conditions of the Late Pleistocene and Holocene according to the results of chironomid analysis of sediments from Medvedevskoe Lake (Karelian Isthmus). Dokl. Earth Sci..

[376] Winkler MG, Swain AM, Kutzbach JE (1986). Middle Holocene Dry Period in the northern midwestern United States: Lake levels and pollen stratigraphy. Quat. Res.

[377] Van Zeist, W. A paleobotanical study of some bogs in western Brittany (Finistere), France. *Palaeohistoria* **10**, 157–180 (1964).

[378] Harbert RS, Nixon KC (2018). Quantitative Late Quaternary Climate Reconstruction from Plant Macrofossil Communities in Western North America. Open Quat.

[379] Affolter, S. *et al*. Central Europe temperature constrained by speleothem fluid inclusion water isotopes over the past 14,000 years. *Sci*. *Adv*. **5**, eaav3809 (2019). 10.1126/sciadv.aav3809 PMC6551184 31183398

[380] Lundeen Z, Brunelle A, Burns SJ, Polyak V, Asmerom Y (2013). A speleothem record of Holocene paleoclimate from the northern Wasatch Mountains, southeast Idaho, USA. Quat. Int.

[381] Sarv A, Il’ves EO (1975). Ueber das Alter der holozaenen Ablagerungen im Muendungsgebiet des Flusses Emajogi (Saviku). Keem. Geol.

[382] Jara IA, Newnham RM, Alloway BV, Wilmshurst JM, Rees AB (2017). Pollen-based temperature and precipitation records of the past 14,600 years in northern New Zealand (37°S) and their linkages with the Southern Hemisphere atmospheric circulation. The Holocene.

[383] Gaudreau, D. C. *Late-Quaternary vegetational history of the northeast: Paleoecological implications of topographic patterns in pollen distributions*. (Yale University, 1986).

[384] Bostwick, L. G. *An environmental framework for cultural change in Maine: Pollen influx and percentage diagrams from Monhegan Island*. (University of Maine, 1978).

[385] Laird KR, Fritz SC, Grimm EC, Mueller PG (1996). Century scale paleoclimatic reconstruction from Moon Lake, a closed-basin lake in the northern Great Plains. Limnol. Oceanogr..

[386] Hahne J (1992). Untersuchungen zur spät- und postglazialen Vegetationsgeschichte im nordöstlichen Bayern (Bayerisches Vogtland, Fichtelgebirge, Steinwald). Flora.

[387] Clegg BF (2010). Six millennia of summer temperature variation based on midge analysis of lake sediments from Alaska. Quat. Sci. Rev..

[388] Harada, N., Ahagon, N., Uchida, M. & Murayama, M. Northward and southward migrations of frontal zones during the past 40 kyr in the Kuroshio-Oyashio transition area. *Geochem*. *Geophys*. *Geosystems* **5**, GC000740 (2004).

[389] Werner K, Spielhagen RF, Bauch D, Hass HC, Kandiano E (2013). Atlantic Water advection versus sea-ice advances in the eastern Fram Strait during the last 9 ka: Multiproxy evidence for a two-phase Holocene. Paleoceanography.

[390] Werner K (2016). Holocene sea subsurface and surface water masses in the Fram Strait – Comparisons of temperature and sea-ice reconstructions. Quat. Sci. Rev..

[391] Ouellet-Bernier M-M, de Vernal A, Hillaire-Marcel C, Moros M (2014). Paleoceanographic changes in the Disko Bugt area, West Greenland, during the Holocene. The Holocene.

[392] Hertzberg JE (2016). Comparison of eastern tropical Pacific TEX86 and *Globigerinoides ruber* Mg/Ca derived sea surface temperatures: Insights from the Holocene and Last Glacial Maximum. Earth Planet. Sci. Lett..

[393] Marchitto TM, Muscheler R, Ortiz JD, Carriquiry JD, van Geen A (2010). Dynamical response of the tropical Pacific Ocean to solar forcing during the Early Holocene. Science.

[394] Janssen CR (1968). Myrtle Lake: A late- and post-glacial pollen diagram from northern Minnesota. Can. J. Bot..

[395] Duplessy JC (1992). Changes in surface salinity of the North Atlantic Ocean during the last deglaciation. Nature.

[396] Willemse NW, Törnqvist TE (1999). Holocene century-scale temperature variability from West Greenland lake records. Geology.

[397] Bertrand S (2017). Postglacial fluctuations of Cordillera Darwin glaciers (southernmost Patagonia) reconstructed from Almirantazgo fjord sediments. Quat. Sci. Rev..

[398] Thompson LG (1995). Late Glacial Stage and Holocene Tropical Ice Core Records from Huascaran, Peru. Science.

[399] North Greenland Ice Core Project members (2004). High-resolution record of Northern Hemisphere climate extending into the last interglacial period. Nature.

[400] Andreev A (2004). Holocene paleoenvironmental records from Nikolay Lake, Lena River Delta, Arctic Russia. Palaeogeogr. Palaeoclimatol. Palaeoecol.

[401] Huguet, C., Kim, J.-H., Sinninghe Damsté, J. S. & Schouten, S. Reconstruction of sea surface temperature variations in the Arabian Sea over the last 23 kyr using organic proxies (TEX86 and U37 K′). *Paleoceanography* **21** (2006).

[402] Bigler C, Barnekow L, Heinrichs ML, Hall RI (2006). Holocene environmental history of Lake Vuolep Njakajaure (Abisko National Park, northern Sweden) reconstructed using biological proxy indicators. Veg. Hist. Archaeobotany.

[403] Larocque I (2004). Holocene temperature estimates and chironomid community composition in the Abisko Valley, northern Sweden. Quat. Sci. Rev..

[404] Gkinis V, Simonsen SB, Buchardt SL, White JWC, Vinther BM (2014). Water isotope diffusion rates from the NorthGRIP ice core for the last 16,000 years – Glaciological and paleoclimatic implications. Earth Planet. Sci. Lett..

[405] Whitehead DR, Crisman TL (1978). Paleolimnological studies of small New England (USA) ponds. Part I. Late-glacial and postglacial trophic oscillations. Pol. Arch. Hydrobiol..

[406] Rösch M (1995). Geschichte des Nussbaumer Sees aus botanisch-ökologischer Sicht. Naturmonographie Die Nussbaumer Seen. Schriftenreihe der Kartause Ittingen.

[407] Keigwin, L. D., Sachs, J. P., Rosenthal, Y. & Boyle, E. A. The 8200 year B.P. event in the slope water system, western subpolar North Atlantic. *Paleoceanography* **20**, PA001074 (2005).

[408] Sachs, J. P. Cooling of Northwest Atlantic slope waters during the Holocene. *Geophys*. *Res*. *Lett*. **34** (2007).

[409] Schmidt MW, Spero HJ, Lea DW (2004). Links between salinity variation in the Caribbean and North Atlantic thermohaline circulation. Nature.

[410] Barron, J. A., Heusser, L., Herbert, T. & Lyle, M. High-resolution climatic evolution of coastal northern California during the past 16,000 years. *Paleoceanography* **18**, PA000768 (2003).

[411] Kim, J.-H., Schneider, R. R., Mulitza, S. & Müller, P. J. Reconstruction of SE trade-wind intensity based on sea-surface temperature gradients in the southeast Atlantic over the last 25 kyr. *Geophys*. *Res*. *Lett*. **30**, GL017557 (2003).

[412] Farmer, E. C., deMenocal, P. B. & Marchitto, T. M. Holocene and deglacial ocean temperature variability in the Benguela upwelling region: Implications for low-latitude atmospheric circulation. *Paleoceanography* **20**, PA001049 (2005).

[413] Shevenell AE, Ingalls AE, Domack EW, Kelly C (2011). Holocene Southern Ocean surface temperature variability west of the Antarctic Peninsula. Nature.

[414] Zhao M, Beveridge NAS, Shackleton NJ, Sarnthein M, Eglinton G (1995). Molecular stratigraphy of cores off northwest Africa: Sea surface temperature history over the last 80 Ka. Paleoceanography.

[415] Came RE, Oppo DW, McManus JF (2007). Amplitude and timing of temperature and salinity variability in the subpolar North Atlantic over the past 10 ky. Geology.

[416] deMenocal P (2000). Coherent high- and low-latitude climate variability during the Holocene Warm Period. Science.

[417] Self AE (2015). The relative influences of climate and volcanic activity on Holocene lake development inferred from a mountain lake in central Kamchatka. Glob. Planet. Change.

[418] Ersek V, Clark PU, Mix AC, Cheng H, Lawrence Edwards R (2012). Holocene winter climate variability in mid-latitude western North America. Nat. Commun..

[419] Watson CS (1996). The vegetational history of the northern Apennines, Italy: Information from three new sequences and a review of regional vegetational change. J. Biogeogr..

[420] Milecka, K. & Szeroczynska, K. Tymczasowa informacja o paleoekologii i paleolimnologii Jeziora Ostrowite na podstawie glebokowodnego rdzenia (z SW czesci zbionika). in *Park Narodowy ‘Bory Tucholskie’*. *[National Park ‘Bory Tucholskie]* (eds. Banaszak, J. & Tobolski, K.) 61–74 (PNBT, 2002).

[421] Sejrup HP, Haflidason H, Andrews JT (2011). A Holocene North Atlantic SST record and regional climate variability. Quat. Sci. Rev..

[422] Tierney JE, Pausata FSR, deMenocal P (2016). Deglacial Indian monsoon failure and North Atlantic stadials linked by Indian Ocean surface cooling. Nat. Geosci..

[423] de Vernal, A., Hillaire-Marcel, C. & Darby, D. A. Variability of sea ice cover in the Chukchi Sea (western Arctic Ocean) during the Holocene. *Paleoceanography* **20** (2005).

[424] Waller MP (1993). Flandrian vegetational history of southeastern England. Pollen data from Pannel Bridge, East Sussex. New Phytologist.

[425] Lynch EA (1998). Origin of a park-forest vegetation mosaic in the Wind River Range, Wyoming. Ecology.

[426] Neil K, Gajewski K, Betts M (2014). Human-ecosystem interactions in relation to Holocene environmental change in Port Joli Harbour, southwestern Nova Scotia, Canada. Quat. Res.

[427] Chang F, Li T, Xiong Z, Xu Z (2015). Evidence for sea level and monsoonally driven variations in terrigenous input to the northern East China Sea during the last 24.3 ka. Paleoceanography.

[428] Minoshima K, Kawahata H, Ikehara K (2007). Changes in biological production in the mixed water region (MWR) of the northwestern North Pacific during the last 27 kyr. Palaeogeogr. Palaeoclimatol. Palaeoecol.

[429] Novenko EY (2015). The Holocene paleoenvironmental history of central European Russia reconstructed from pollen, plant macrofossil, and testate amoeba analyses of the Klukva Peatland, Tula Region. Quat. Res.

[430] Andrén E (2015). Holocene climate and environmental change in north-eastern Kamchatka (Russian Far East), inferred from a multi-proxy study of lake sediments. Glob. Planet. Change.

[431] Lim S, Chase BM, Chevalier M, Reimer PJ (2016). 50,000 years of vegetation and climate change in the southern Namib Desert, Pella, South Africa. Palaeogeogr. Palaeoclimatol. Palaeoecol.

[432] Fisher DA (1998). Penny Ice Cap cores, Baffin Island, Canada, and the Wisconsinan Foxe Dome connection: Two states of Hudson Bay ice cover. Science.

[433] Boyko-Diakonow, M. & Terasmae, J. Palynology of Holocene sediments in Perch Lake, Chalk River, Ontario. in *Hydrological Studies on a Small Basin on the Canadian Shield: A Final Summary of the Perch Lake Evaporation Study 1965–1975* (ed. Barry, P. J.) 189–220 (Energy Can. Ltd., 1975).

[434] Brown KJ, Hebda RJ (2002). Origin, development, and dynamics of coastal temperate conifer rainforests of southern Vancouver Island, Canada. Can. J. For. Res.

[435] Voronina E, Polyak L, Vernal AD, Peyron O (2001). Holocene variations of sea-surface conditions in the southeastern Barents Sea, reconstructed from dinoflagellate cyst assemblages. J. Quat. Sci.

[436] Lea DW (2003). Synchroneity of tropical and high-latitude Atlantic temperatures over the Last Glacial Termination. Science.

[437] Mosley-Thompson, E. Holocene climate changes recorded in an East Antarctica ice core. in *Climatic Variations and Forcing Mechanisms of the Last 2000* Years (eds. Jones, P. D., Bradley, R. S. & Jouzel, J.) 263–279 (Springer Berlin Heidelberg, 1996).

[438] Swain, P. C. *The development of some bogs in eastern Minnesota*. (University of Minnesota, 1979).

[439] Warner BG, Tolonen K, Tolonen M (1991). A postglacial history of vegetation and bog formation at Point Escuminac, New Brunswick. Can. J. Earth Sci..

[440] Constantin S, Bojar A-V, Lauritzen S-E, Lundberg J (2007). Holocene and Late Pleistocene climate in the sub-Mediterranean continental environment: A speleothem record from Poleva Cave (Southern Carpathians, Romania). Palaeogeogr. Palaeoclimatol. Palaeoecol.

[441] Shafer, D. S. *The timing of late Quaternary monsoon precipitation maxima in the southwest United States*. (University of Arizona, 1989).

[442] Massaferro J, Larocque-Tobler I (2013). Using a newly developed chironomid transfer function for reconstructing mean annual air temperature at Lake Potrok Aike, Patagonia, Argentina. Ecol. Indic..

[443] Mary Y (2017). Changes in Holocene meridional circulation and poleward Atlantic flow: The Bay of Biscay as a nodal point. Clim. Past.

[444] Shotyk W, Cheburkin AK, Appleby PG, Fankhauser A, Kramers JD (1997). Lead in three peat bog profiles, Jura Mountains, Switzerland: Enrichment factors, isotopic composition, and chronology of atmospheric deposition. Water. Air. Soil Pollut.

[445] Risebrobakken, B. *et al*. Early Holocene temperature variability in the Nordic Seas: The role of oceanic heat advection versus changes in orbital forcing. *Paleoceanography* **26**, PA002117 (2011).

[446] Penalba, M. C. *Dynamique de vegetation Tardiglaciaire et Holocene du centre-nord de l’Espagne d’apres l’analyse pollinique*. (Universite d’Aix-Marseille, 1989).

[447] van den Bos V (2018). Holocene temperature, humidity and seasonality in northern New Zealand linked to Southern Hemisphere summer insolation. Quat. Sci. Rev..

[448] Obidowicz A (1993). Wahania gornej granicy lasu w poznym plejstocenie i holocenie w Tatrach [Fluctuations of the forest timberline in the Tatra Mountains during the last 12 000 years]. Dok. Geogr.

[449] Kaplan MR, Wolfe AP, Miller GH (2002). Holocene Environmental Variability in Southern Greenland Inferred from Lake Sediments. Quat. Res.

[450] Wooller MJ (2012). An ~11,200 year paleolimnological perspective for emerging archaeological findings at Quartz Lake, Alaska. J. Paleolimnol..

[451] Seppä H, Poska A (2004). Holocene annual mean temperature changes in Estonia and their relationship to solar insolation and atmospheric circulation patterns. Quat. Res..

[452] Pirrus, R., Rouk, A. M. & Liiva, A. Geology and stratigraphy of the reference site of Lake Raigastvere in Saadjaerv drumlin field. In *Palaeohydrology of the temperate zone* II. Lakes. (eds. Raukas, A. & Saarse, L.) 101–122 (1987).

[453] Brubaker LB, Garfinkel HL, Edwards ME (1983). A Late Wisconsin and Holocene Vegetation History from the central Brooks Range: Implications for Alaskan Palaeoecology. Quat. Res.

[454] Fall, P. L. *Vegetation dynamics in the southern Rocky Mountains: Late Pleistocene and Holocene timberline fluctuations*. (University of Arizona, 1988).

[455] Thornalley, D. J. R., Elderfield, H. & McCave, I. N. Intermediate and deep water paleoceanography of the northern North Atlantic over the past 21,000 years. *Paleoceanography* **25** (2010).

[456] Poore, R. Z., Dowsett, H. J., Verardo, S. & Quinn, T. M. Millennial- to century-scale variability in Gulf of Mexico Holocene climate records. *Paleoceanography* **18** (2003).

[457] Arbuszewski JA, deMenocal PB, Cléroux C, Bradtmiller L, Mix A (2013). Meridional shifts of the Atlantic Intertropical Convergence Zone since the Last Glacial Maximum. Nat. Geosci..

[458] Overpeck J, Anderson D, Trumbore S, Prell W (1996). The southwest Indian Monsoon over the last 18 000 years. Clim. Dyn.

[459] Maher LJ (1972). Absolute pollen diagram of Redrock Lake, Boulder County, Colorado. Quat. Res.

[460] Almquist-Jacobson H, Almendinger JE, Hobbie S (1992). Influence of terrestrial vegetation on sediment-forming processes in kettle lakes of west-central Minnesota. Quat. Res.

[461] Johnsen SJ (1992). Irregular glacial interstadials recorded in a new Greenland ice core. Nature.

[462] Moffa‐Sanchez P, Rosenthal Y, Babila TL, Mohtadi M, Zhang X (2019). Temperature evolution of the Indo‐Pacific Warm Pool over the Holocene and the last deglaciation. Paleoceanogr. Paleoclimatology.

[463] Hicks, S. Problems and possibilities in correlating historical/archaeological and pollen-analytical evidence in a northern boreal environment: An example from Kuusamo, Finland. *Fennosc*. *Archaeol*. **II** (1985).

[464] Kaislahti Tillman P (2010). Long-term climate variability in continental subarctic Canada: A 6200-year record derived from stable isotopes in peat. Palaeogeogr. Palaeoclimatol. Palaeoecol.

[465] Loomis SE, Russell JM, Ladd B, Street-Perrott FA, Sinninghe Damsté JS (2012). Calibration and application of the branched GDGT temperature proxy on East African lake sediments. Earth Planet. Sci. Lett..

[466] Wick L, van Leeuwen JFN, van der Knaap WO, Lotter AF (2003). Holocene vegetation development in the catchment of Sägistalsee (1935 m asl), a small lake in the Swiss Alps. J. Paleolimnol.

[467] Rao Z (2019). Long-term summer warming trend during the Holocene in central Asia indicated by alpine peat α-cellulose δ13C record. Quat. Sci. Rev..

[468] Bernard, J. *Paleoenvironnement du Pays de Retz et du marais Breton-Vendeen*. (Universite de Nantes, 1996).

[469] Richard PJH (1971). Two pollen diagrams from the Quebec City area, Canada. Pollen Spores.

[470] Ilyashuk EA, Koinig KA, Heiri O, Ilyashuk BP, Psenner R (2011). Holocene temperature variations at a high-altitude site in the eastern Alps: A chironomid record from Schwarzsee ob Sölden, Austria. Quat. Sci. Rev..

[471] Lasher GE (2017). Holocene temperatures and isotopes of precipitation in Northwest Greenland recorded in lacustrine organic materials. Quat. Sci. Rev..

[472] Stebich M (2015). Holocene vegetation and climate dynamics of NE China based on the pollen record from Sihailongwan Maar Lake. Quat. Sci. Rev..

[473] Das, S. B. & Alley, R. B. Rise in frequency of surface melting at Siple Dome through the Holocene: Evidence for increasing marine influence on the climate of West Antarctica. *J*. *Geophys*. *Res*. **113** (2008).

[474] Rosén P, Segerström U, Eriksson L, Renberg I (2003). Do diatom, chironomid, and pollen records consistently infer Holocene July air temperature? A comparison using sediment cores from four alpine lakes in northern Sweden. Arct. Antarct. Alp. Res..

[475] Saraswat R, Lea DW, Nigam R, Mackensen A, Naik DK (2013). Deglaciation in the tropical Indian Ocean driven by interplay between the regional monsoon and global teleconnections. Earth Planet. Sci. Lett..

[476] Litt T (2001). Correlation and synchronisation of Lateglacial continental sequences in northern central Europe based on annually laminated lacustrine sediments. Quat. Sci. Rev..

[477] Smith AG, Goddard IC (1991). A 12 500 year record of vegetational history at Sluggan Bog, Co. Antrim, N. Ireland (incorporating a pollen zone scheme for the non-specialist). New Phytol..

[478] Tiwari M, Nagoji SS, Ganeshram RS (2015). Multi-centennial scale SST and Indian summer monsoon precipitation variability since the mid-Holocene and its nonlinear response to solar activity. The Holocene.

[479] Barrows TT, Lehman SJ, Fifield LK, De Deckker P (2007). Absence of cooling in New Zealand and the adjacent ocean during the Younger Dryas chronozone. Science.

[480] Mohtadi M (2014). North Atlantic forcing of tropical Indian Ocean climate. Nature.

[481] Sirocko F (2000). Processes controlling trace element geochemistry of Arabian Sea sediments during the last 25,000 years. Glob. Planet. Change.

[482] Doose-Rolinski H, Rogalla U, Scheeder G, Lückge A, von Rad U (2001). High-resolution temperature and evaporation changes during the Late Holocene in the northeastern Arabian Sea. Paleoceanography.

[483] Staubwasser, M., Sirocko, F., Grootes, P. M. & Segl, M. Climate change at the 4.2 ka BP termination of the Indus valley civilization and Holocene south Asian monsoon variability. *Geophys*. *Res*. *Lett*. **30**, GL016822 (2003).

[484] Shala S (2017). Comparison of quantitative Holocene temperature reconstructions using multiple proxies from a northern boreal lake. The Holocene.

[485] Chevalier M, Chase BM (2015). Southeast African records reveal a coherent shift from high- to low-latitude forcing mechanisms along the east African margin across last glacial–interglacial transition. Quat. Sci. Rev..

[486] Williams PW, King DNT, Zhao J-X, Collerson KD (2005). Late Pleistocene to Holocene composite speleothem 18O and 13C chronologies from South Island, New Zealand—did a global Younger Dryas really exist?. Earth Planet. Sci. Lett..

[487] Ohlwein C, Wahl ER (2012). Review of probabilistic pollen-climate transfer methods. Quat. Sci. Rev..

[488] Lauritzen S-E, Lundberg J (1999). Calibration of the speleothem delta function: An absolute temperature record for the Holocene in northern Norway. The Holocene.

[489] Adams JK, Finkelstein SA (2010). Watershed-scale reconstruction of middle and late Holocene paleoenvironmental changes on Melville Peninsula, Nunavut, Canada. Quat. Sci. Rev..

[490] Hammarlund D (2004). Palaeolimnological and sedimentary responses to Holocene forest retreat in the Scandes Mountains, west-central Sweden. The Holocene.

[491] Fohlmeister J, Vollweiler N, Spötl C, Mangini A (2013). COMNISPA II: Update of a mid-European isotope climate record, 11 ka to present. The Holocene.

[492] Novenko, E. Y., Tsyganov, A. N. & Olchev, A. V. Palaeoecological data as a tool to predict possible future vegetation changes in the boreal forest zone of European Russia: A case study from the Central Forest Biosphere Reserve. *IOP Conf*. *Ser*. *Earth Environ*. *Sci*. **107**, 012104 (2018).

[493] Andresen CS, Björck S, Rundgren M, Conley DJ, Jessen C (2008). Rapid Holocene climate changes in the North Atlantic: Evidence from lake sediments from the Faroe Islands. Boreas.

[494] Bringué M, Rochon A (2012). Late Holocene paleoceanography and climate variability over the Mackenzie Slope (Beaufort Sea, Canadian. Arctic). Mar. Geol.

[495] Reinemann SA, Porinchu DF, Bloom AM, Mark BG, Box JE (2009). A multi-proxy paleolimnological reconstruction of Holocene climate conditions in the Great Basin, United States. Quat. Res.

[496] Lemmen J, Lacourse T (2018). Fossil chironomid assemblages and inferred summer temperatures for the past 14,000 years from a low-elevation lake in Pacific Canada. J. Paleolimnol..

[497] Tonkov S, Bozilova E, Jungner H (2009). 7. Mire Straldza (Southeastern Bulgaria): Late Holocene vegetation history. Grana.

[498] Bard E (2000). Hydrological impact of Heinrich events in the subtropical northeast Atlantic. Science.

[499] Elovicheva, Y. K. & Bogdel, I. I. Novye razrezy golosena Belarusi [New Holocene sections of Byelorussia]. in: *Geologicheskoe stroenie osadochnoi tolshchi Belorussii [Geological composition of sedimentary sequence of Byelorussia]* (eds. Kuznetsov, A., Ropot, V. F., & Elovicheva1, Ia. K.) 141–169 (Nauka i Tekhnika, Minsk, 1985).

[500] Rigual-Hernández AS (2017). Svalbard ice-sheet decay after the Last Glacial Maximum: New insights from micropalaeontological and organic biomarker paleoceanographical reconstructions. Palaeogeogr. Palaeoclimatol. Palaeoecol.

[501] Bjune AE, Birks HJB (2008). Holocene vegetation dynamics and inferred climate changes at Svanåvatnet, Mo i Rana, northern Norway. Boreas.

[502] Luoto TP (2018). Synchronized proxy-based temperature reconstructions reveal mid- to late Holocene climate oscillations in high arctic Svalbard. J. Quat. Sci.

[503] Behre, K. E. & Kucan, D. Die Reflektion archaeologisch bekannter Siedlungen in Pollendiagrammen verschiedener Entfernung. - Beispiele aus der Siedlungskammer Floegeln, Nordwestdeutschland. in *Anthropogenic indicators in pollen diagrams* (ed. Behre, K. E.) 95–114 (Balkema, Rotterdam, The Netherlands, 1986).

[504] Makohonienko, M. *Przyrodnicza Historia Gniezna . [Natural History of Gniezno]*. (Homini, Bydogoszcz-Poznan, 2000).

[505] Szczepanek K (1989). Type region P-c: Low Beskidy Mountains. Acta Palaeobot.

[506] Mezgec K (2017). Holocene sea ice variability driven by wind and polynya efficiency in the Ross Sea. Nat. Commun..

[507] Langdon PG, Barber KE, Lomas-Clarke (previously Morriss) SH (2004). Reconstructing climate and environmental change in northern England through chironomid and pollen analyses: Evidence from Talkin Tarn, Cumbria. J. Paleolimnol..

[508] Loomis SE, Russell JM, Lamb HF (2015). Northeast African temperature variability since the Late Pleistocene. Palaeogeogr. Palaeoclimatol. Palaeoecol.

[509] Tierney JE (2008). Northern Hemisphere controls on tropical southeast African climate during the past 60,000 years. Science.

[510] Anderson L, Abbott MB, Finney BP (2001). Holocene climate inferred from oxygen isotope ratios in lake sediments, central Brooks Range, Alaska. Quat. Res.

[511] Harmata K (1987). Late-Glacial and Holocene history of vegetation at Roztoki and Tarnowiec near Jaslo (Jaslo-Sanok Depression). Acta Palaeobot.

[512] Tóth M (2015). Chironomid-inferred Holocene temperature changes in the South Carpathians (Romania). The Holocene.

[513] Diaconu A-C (2017). How warm? How wet? Hydroclimate reconstruction of the past 7500 years in northern Carpathians, Romania. Palaeogeogr. Palaeoclimatol. Palaeoecol.

[514] Nazarova L, Lüpfert H, Subetto D, Pestryakova L, Diekmann B (2013). Holocene climate conditions in central Yakutia (Eastern Siberia) inferred from sediment composition and fossil chironomids of Lake Temje. Quat. Int.

[515] Zhang E (2017). Holocene high-resolution quantitative summer temperature reconstruction based on subfossil chironomids from the southeast margin of the Qinghai-Tibetan Plateau. Quat. Sci. Rev..

[516] Hammarlund D, Barnekow L, Birks HJB, Buchardt B, Edwards TWD (2002). Holocene changes in atmospheric circulation recorded in the oxygen-isotope stratigraphy of lacustrine carbonates from northern Sweden. The Holocene.

[517] Cheddadi R, Lamb HF, Guiot J, van der Kaars S (1998). Holocene climatic change in Morocco: A quantitative reconstruction from pollen data. Clim. Dyn.

[518] Nielsen SHH, Koç N, Crosta X (2004). Holocene climate in the Atlantic sector of the Southern Ocean: Controlled by insolation or oceanic circulation?. Geology.

[519] Barron JA, Bukry D, Heusser LE, Addison JA, Alexander CR (2018). High-resolution climate of the past ~7300 years of coastal northernmost California: Results from diatoms, silicoflagellates, and pollen. Quat. Int.

[520] Paus A, Velle G, Berge J (2011). The Lateglacial and early Holocene vegetation and environment in the Dovre mountains, central Norway, as signalled in two Lateglacial nunatak lakes. Quat. Sci. Rev..

[521] Grudd H (2002). A 7400-year tree-ring chronology in northern Swedish Lapland: Natural climatic variability expressed on annual to millennial timescales. The Holocene.

[522] Seppä H, Nyman M, Korhola A, Weckström J (2002). Changes of treelines and alpine vegetation in relation to post-glacial climate dynamics in northern Fennoscandia based on pollen and chironomid records. J. Quat. Sci.

[523] Comtois P (1982). Histoire Holocène du climat et de la végétation à Lanoraie (Québec). Can. J. Earth Sci..

[524] Lea DW (2006). Paleoclimate history of Galápagos surface waters over the last 135,000 yr. Quat. Sci. Rev..

[525] Dubois, N., Kienast, M., Normandeau, C. & Herbert, T. D. Eastern equatorial Pacific cold tongue during the Last Glacial Maximum as seen from alkenone paleothermometry. *Paleoceanography* **24**, PA001781 (2009).

[526] Antonsson K, Seppä H (2007). Holocene temperatures in Bohuslän, southwest Sweden: A quantitative reconstruction from fossil pollen data. Boreas.

[527] Bjune AE, Bakke J, Nesje A, Birks HJB (2005). Holocene mean July temperature and winter precipitation in western Norway inferred from palynological and glaciological lake-sediment proxies. The Holocene.

[528] Klitgaard-Kristensen D, Sejrup HP, Haflidason H (2001). The last 18 kyr fluctuations in Norwegian sea surface conditions and implications for the magnitude of climatic change: Evidence from the North Sea. Paleoceanography.

[529] Irvine F, Cwynar LC, Vermaire JC, Rees ABH (2012). Midge-inferred temperature reconstructions and vegetation change over the last ~15,000 years from Trout Lake, northern Yukon Territory, eastern Beringia. J. Paleolimnol..

[530] Rodrigo-Gámiz M, Martínez-Ruiz F, Rampen SW, Schouten S, Sinninghe Damsté JS (2014). Sea surface temperature variations in the western Mediterranean Sea over the last 20 kyr: A dual-organic proxy (U K′ 37 and LDI) approach. Paleoceanography.

[531] Berke MA, Johnson TC, Werne JP, Schouten S, Sinninghe Damsté JS (2012). A mid-Holocene thermal maximum at the end of the African Humid Period. Earth Planet. Sci. Lett..

[532] Nazarova L, de Hoog V, Hoff U, Dirksen O, Diekmann B (2013). Late Holocene climate and environmental changes in Kamchatka inferred from the subfossil chironomid record. Quat. Sci. Rev..

[533] Verbruggen, C. *Paleoecologische en palynlogische benadering van enkele bekende historisch-geografisch problemen in Vlaaderen. Bronnen voor de historisch geografie van Belgie* 487–497 (Handelingen van het Colloquium te Brussel, 1979).

[534] Bunbury J, Gajewski K (2009). Postglacial climates inferred from a lake at treeline, southwest Yukon Territory, Canada. Quat. Sci. Rev..

[535] Van Zeist W, Woldring H (1978). A postglacial pollen diagram from Lake Van in East Anatolia. Rev. Palaeobot. Palynol..

[536] Holmes N, Langdon PG, Caseldine C, Brooks SJ, Birks HJB (2011). Merging chironomid training sets: Implications for palaeoclimate reconstructions. Quat. Sci. Rev..

[537] Peichlova, M. *Historie vegetace Broumovska [Vegetation history of the Broumovska region]*. (Academy of Science CR, 1979).

[538] Balascio NL, Bradley RS (2012). Evaluating Holocene climate change in northern Norway using sediment records from two contrasting lake systems. J. Paleolimnol..

[539] Schmidt MW (2012). Impact of abrupt deglacial climate change on tropical Atlantic subsurface temperatures. Proc. Natl. Acad. Sci.

[540] Koutavas, A. & Lynch-Stieglitz, J. Glacial-interglacial dynamics of the eastern equatorial Pacific cold tongue-Intertropical Convergence Zone system reconstructed from oxygen isotope records. *Paleoceanography* **18** (2003).

[541] Vimeux F, Cuffey KM, Jouzel J (2002). New insights into Southern Hemisphere temperature changes from Vostok ice cores using deuterium excess correction. Earth Planet. Sci. Lett..

[542] Berntsson A, Rosqvist GC, Velle G (2014). Late-Holocene temperature and precipitation changes in Vindelfjällen, mid-western Swedish Lapland, inferred from chironomid and geochemical data. The Holocene.

[543] Heinrichs M, Barnekow L, Rosenberg S (2006). A comparison of chironomid biostratigraphy from Lake Vuolep Njakajaure with vegetation, lake-level, and climate changes in Abisko National Park, Sweden. J. Paleolimnol..

[544] Bigler C, Larocque I, Peglar SM, Birks HJB, Hall RI (2002). Quantitative multiproxy assessment of long-term patterns of Holocene environmental change from a small lake near Abisko, northern Sweden. The Holocene.

[545] Doerfler, W. Pollenanalytische Untersuchungen zur Vegetations- und Siedlungsgeschichte im Sueden des Landkreises Cuxhaven, Niedersachsen. *Probl*. *Kuestenforschung Im Suedlichen Nord*. **17** (1989).

[546] Oeschger H (1980). 14C and other parameters during the Younger Dryas cold phase. Radiocarbon.

[547] Fegyveresi JM (2016). Five millennia of surface temperatures and ice core bubble characteristics from the WAIS Divide deep core, West Antarctica. Paleoceanography.

[548] Cuffey KM (2016). Deglacial temperature history of West Antarctica. Proc. Natl. Acad. Sci..

[549] Levy LB, Kaufman DS, Werner A (2004). Holocene glacier fluctuations, Waskey Lake, northeastern Ahklun Mountains, southwestern Alaska. The Holocene.

[550] Huettemann, H. & Bortenschlager, S. Beitraege zur Vegetationsgeschichte Tirols VI: Riesengebirge, Hohe Tatra - Zillertal, Kuehtai. *Ber Nat-Med Ver*. *Innsbr*. **74** (1987).

[551] Willard DA, Weimer LM, Riegel WL (2001). Pollen assemblages as paleoenvironmental proxies in the Florida Everglades. Rev. Palaeobot. Palynol..

[552] Krause TR, Russell JM, Zhang R, Williams JW, Jackson ST (2019). Late Quaternary vegetation, climate, and fire history of the Southeast Atlantic Coastal Plain based on a 30,000-yr multi-proxy record from White Pond, South Carolina, USA. Quat. Res.

[553] Waller, M. P. *The Fenland Project, Number 9: Flandrian Environmental Change in Fenland*. (East Anglian Archaeology Monograph, no. 70, 1994).

[554] Rösch, M. Botanical evidence for prehistoric and medieval land use in the Black Forest. in *Medieval Rural Settlement in Marginal Landscapes* (eds. Klápšte, J. & Sommer, P.) vol. 7, 335–343 (Brepols Publishers, 2009).

[555] Kiefer T, McCave IN, Elderfield H (2006). Antarctic control on tropical Indian Ocean sea surface temperature and hydrography. Geophys. Res. Lett..

[556] Chase M, Bleskie C, Walker IR, Gavin DG, Hu FS (2008). Midge-inferred Holocene summer temperatures in Southeastern British Columbia, Canada. Palaeogeogr. Palaeoclimatol. Palaeoecol.

[557] Truc L (2013). Quantification of climate change for the last 20,000 years from Wonderkrater, South Africa: Implications for the long-term dynamics of the Intertropical Convergence Zone. Palaeogeogr. Palaeoclimatol. Palaeoecol.

[558] Pawlikowski, M., Ralska-Jasiewiczowa, M., Schoenborn, W., Stupnicka, E. & Szeroczynska, K. Woryty near Gietrzwald, Olsztyn Lake District, NE Poland - vegetational history and lake development during the last 12,000 years. *Acta Palaeobot*. **22**, 85–116 (1982).

[559] Wu D (2018). Decoupled early Holocene summer temperature and monsoon precipitation in southwest China. Quat. Sci. Rev..

[560] Leipe C, Kito N, Sakaguchi Y, Tarasov PE (2013). Vegetation and climate history of northern Japan inferred from the 5500-year pollen record from the Oshima Peninsula, SW Hokkaido. Quat. Int.

[561] Roberts SJ (2017). Past penguin colony responses to explosive volcanism on the Antarctic Peninsula. Nat. Commun..

[562] Seppä H, MacDonald GM, Birks HJB, Gervais BR, Snyder JA (2008). Late-Quaternary summer temperature changes in the northern-European tree-line region. Quat. Res.

[563] Huttenen A (1990). Vegetation and palaeoecology of a bog complex in southern Finland. Aquilo Ser. Bot.

[564] Płóciennik M, Self A, Birks HJB, Brooks SJ (2011). Chironomidae (Insecta: Diptera) succession in Żabieniec bog and its palaeo-lake (central Poland) through the Late Weichselian and Holocene. Palaeogeogr. Palaeoclimatol. Palaeoecol..

[565] Bezusko, L. G., Kajutkina, T. M. & Kovalyukh, N. N. *YIII s’ezd Ukrainskovo botanicheskogo obschestva [New data of Allerod vegetation of Ukraine]*. (1992).

[566] Rybníčková E, Rybníček K, Jankovská V (1975). Palaeoecological investigations of buried peat profiles from the Zbudovská blata marshes, Southern Bohemia. Folia Geobot. Phytotaxon..

[567] Schneider L (2019). The impact of proxy selection strategies on a millennium-long ensemble of hydroclimatic records in Monsoon Asia. Quat. Sci. Rev..

[568] Shakun JD (2012). Global warming preceded by increasing carbon dioxide concentrations during the last deglaciation. Nature.

[569] Sommer P (2019). Zenodo.

[570] Boos D (2003). Introduction to the bootstrap world. Stat. Sci..

[571] PAGES 2k Consortium. (2019). Consistent multidecadal variability in global temperature reconstructions and simulations over the Common Era. Nat. Geosci..

[572] Poli P (2016). ERA-20C: An atmospheric reanalysis of the Twentieth Century. J. Clim..

[573] Morice CP, Kennedy JJ, Rayner NA, Jones PD (2012). Quantifying uncertainties in global and regional temperature change using an ensemble of observational estimates: The HadCRUT4 data set. J. Geophys. Res. Atmospheres.

[574] Cowtan K, Way RG (2014). Coverage bias in the HadCRUT4 temperature series and its impact on recent temperature trends: Coverage bias in the HadCRUT4 temperature series. Q. J. R. Meteorol. Soc..

[575] Braconnot P (2012). Evaluation of climate models using palaeoclimatic data. Nat. Clim. Change.

[576] Bradley, R. S. *Paleoclimatology: reconstructing climates of the Quaternary*. (Elsevier, 2015).

[577] Telford RJ (2019). Review and test of reproducibility of subdecadal resolution palaeoenvironmental reconstructions from microfossil assemblages. Quat. Sci. Rev..

[578] Kaufman DS (2016). Holocene climate changes in eastern Beringia (NW North America) – A systematic review of multi-proxy evidence. Quat. Sci. Rev..

[579] Briner JP (2016). Holocene climate change in Arctic Canada and Greenland. Quat. Sci. Rev..

[580] Sejrup HP (2016). North Atlantic-Fennoscandian Holocene climate trends and mechanisms. Quat. Sci. Rev..

[581] Routson C, McKay N, Sommer P, Dätwyler C (2020). Zenodo.

[582] Williams, J. W., Kaufman, D., Newton, A. & von Gunten, L. *Building and Harnessing Open Paleodata*. Past Global Changes Magazine **26**(2) (2018).

