# Publisher Correction: A global database of Holocene paleotemperature records

**DOI:** 10.1038/s41597-020-0515-6

**Published:** 2020-06-15

**Authors:** Darrell Kaufman, Nicholas McKay, Cody Routson, Michael Erb, Basil Davis, Oliver Heiri, Samuel Jaccard, Jessica Tierney, Christoph Dätwyler, Yarrow Axford, Thomas Brussel, Olivier Cartapanis, Brian Chase, Andria Dawson, Anne de Vernal, Stefan Engels, Lukas Jonkers, Jeremiah Marsicek, Paola Moffa-Sánchez, Carrie Morrill, Anais Orsi, Kira Rehfeld, Krystyna Saunders, Philipp S. Sommer, Elizabeth Thomas, Marcela Tonello, Mónika Tóth, Richard Vachula, Andrei Andreev, Sebastien Bertrand, Boris Biskaborn, Manuel Bringué, Stephen Brooks, Magaly Caniupán, Manuel Chevalier, Les Cwynar, Julien Emile-Geay, John Fegyveresi, Angelica Feurdean, Walter Finsinger, Marie-Claude Fortin, Louise Foster, Mathew Fox, Konrad Gajewski, Martin Grosjean, Sonja Hausmann, Markus Heinrichs, Naomi Holmes, Boris Ilyashuk, Elena Ilyashuk, Steve Juggins, Deborah Khider, Karin Koinig, Peter Langdon, Isabelle Larocque-Tobler, Jianyong Li, André Lotter, Tomi Luoto, Anson Mackay, Eniko Magyari, Steven Malevich, Bryan Mark, Julieta Massaferro, Vincent Montade, Larisa Nazarova, Elena Novenko, Petr Pařil, Emma Pearson, Matthew Peros, Reinhard Pienitz, Mateusz Płóciennik, David Porinchu, Aaron Potito, Andrew Rees, Scott Reinemann, Stephen Roberts, Nicolas Rolland, Sakari Salonen, Angela Self, Heikki Seppä, Shyhrete Shala, Jeannine-Marie St-Jacques, Barbara Stenni, Liudmila Syrykh, Pol Tarrats, Karen Taylor, Valerie van den Bos, Gaute Velle, Eugene Wahl, Ian Walker, Janet Wilmshurst, Enlou Zhang, Snezhana Zhilich

**Affiliations:** 1 Northern Arizona University, School of Earth and Sustainability, Flagstaff, AZ 86011 USA; 2 University of Lausanne, Institute of Earth Surface Dynamics, Lausanne, 1015 Switzerland; 3 University of Basel, Department of Environmental Sciences, Basel, 4056 Switzerland; 4 University of Bern, Institute of Geological Sciences and Oeschger Center for Climate Change Research, Bern, CH-3012 Switzerland; 5 University of Arizona, Department of Geosciences, Tucson, AZ 85721 USA; 6 University of Bern, Institute of Geography and Oeschger Centre for Climate Change Research, Bern, 3012 Switzerland; 7 Northwestern University, Department of Earth and Planetary Sciences, Evanston, IL 60208 USA; 8 University of Utah, Department of Geography, Salt Lake City, UT 84112 USA; 9 Université de Montpellier, Centre National de la Recherche Scientifique, Institut des Sciences de l’Evolution, Montpellier, 34095 France; 10 Mount Royal University, Department of General Education, Calgary, T3E6K6 Canada; 11 Université du Québec à Montréal, Geotop-UQAM, Montréal, H3C 3P8 Canada; 12 University of London, Birkbeck, Department of Geography, London, WC1E 7HX UK; 13 University of Bremen, MARUM Center for Marine Environmental Sciences, Bremen, 28359 Germany; 14 University of Wisconsin-Madison, Department of Geoscience, Madison, WI 53706 USA; 15 Durham University, Department of Geography, Durham, DH1 3LE UK; 16 University of Colorado, Cooperative Institute for Research in Environmental Sciences, Boulder, CO 80309 USA; 17 Laboratoire des Sciences du Climat et de l’Environnement, Université Paris-Saclay, Gif sur Yvette, 91191 France; 18 Heidelberg University, Institute of Environmental Physics, Heidelberg, 69221 Germany; 19 Australian Nuclear Science and Technology Organisation, Environment, Lucas Heights, 2234 Australia; 20 Institute for Coastal Research, Helmholtz-Zentrum, Geesthacht, Germany; 21 University at Buffalo, Department of Geology, Buffalo, NY 14206 USA; 22 Universidad Nacional de Mar del Plata, Instituto de Investigaciones Marinas y Costeras, Mar del Plata, 7600 Argentina; 23 Balaton Limnological Institute, Centre for Ecological Research, Tihany, H-8237 Hungary; 24 Brown University, Department of Earth, Environmental and Planetary Sciences, Providence, 2912 USA; 25 Alfred Wegener Institut Helmholtz Centre for Polar and Marine Research, Polar Terrestrial Environmental Systems, Potsdam, 14473 Germany; 26 Ghent University, Renard Centre of Marine Geology, Gent, 9000 Belgium; 27 Natural Resources Canada, Geological Survey of Canada, Calgary, AB T2L 2A7 Canada; 28 Natural History Museum, Department of Life Sciences, London, SW7 5BD UK; 29 University of Concepcion, Department of Oceanography and COPAS Sur-Austral Program, Concepcion, 4030000 Chile; 30 University of New Brunswick, Department of Biology, Fredericton, NB E3B 5A3 Canada; 31 University of Southern California, Department of Earth Sciences, Los Angeles, CA 90089 USA; 32 Goethe University, Department of Physical Geography, Frankfurt am Main, 60438 Germany; 33 University of Ottawa, Ottawa-Carleton Institute of Biology, Ottawa, K1N6N5 Canada; 34 Newcastle University, School of Geography, Politics and Sociology, Newcastle-upon-Tyne, NE17RU UK; 35 British Antarctic Survey, Palaeoenvironments and Ice Sheets, Cambridge, CB3 0ET UK; 36 University of Arizona, School of Anthropology, Tucson, AZ 85721 USA; 37 University of Ottawa, Department of Geography, Environment and Geomatics, Ottawa, K1N6N5 Canada; 38 Aquatica GmbH, Bern, 3007 Switzerland; 39 Okanagan College, Department of Geography and Earth and Environmental Science, Kelowna, V1Y 4X8 Canada; 40 Sheffield Hallam University, Department of the Natural and Built Environment, Sheffield, S1 1WB UK; 41 University of Innsbruck, Department of Ecology, Innsbruck, 6020 Austria; 42 University of Southern California, Information Sciences Institute, Marina Del Rey, CA 90292 USA; 43 University of Southampton, School of Geography and Environmental Science, Southampton, SO17 1BJ UK; 44 The LAKES Institute, Lyss, 3250 Switzerland; 45 Northwest University, China, College of Urban and Environmental Sciences, Xi’an, 710027 China; 46 University of Bern, Palaeoecology, Bern, CH-3013 Switzerland; 47 University of Helsinki, Faculty of Biological and Environmental Sciences, Lahti, 15140 Finland; 48 University College London, Department of Geography, London, WC1E 6BT UK; 49 Eötvös Loránd University, Department of Environmental and Landscape Geography, Budapest, 1117 Hungary; 50 The Ohio State University, Department of Geography and Byrd Polar and Climate Research Center, Columbus, OH 43210 USA; 51 CONICET Argentina, CENAC/APN, Bariloche, RN 8400 Argentina; 52 Potsdam University, Institute of Geosciences, Potsdam, 14476 Germany; 53 Lomonosov Moscow State University, Faculty of Geography, Moscow, 119991 Russia; 54 Masaryk University, Department of Botany and Zoology, Brno, 61137 Czech Republic; 55 Bishop’s University, Department of Environment and Geography, Sherbrooke, Quebec J1M 1Z7 Canada; 56 Université Laval, Department of Geography, Center for Northern Studies, Québec, G1V 0A6 Canada; 57 University of Lodz, Department of Invertebrate Zoology and Hydrobiology, Lodz, 90-237 Poland; 58 University of Georgia, Department of Geography, Athens, GA 30606 USA; 59 National University of Ireland Galway, School of Geography, Archaeology and Irish Studies, Galway, H91 TK33 Ireland; 60 Victoria University of Wellington, School of Geography, Environment and Earth Sciences, Wellington, 6012 New Zealand; 61 Sinclair Community College, Geography Department, Dayton, OH 45402 USA; 62 Fisheries and Ocean Canada, Gulf Fisheries Centre, Moncton, NB E1C 9B6 Canada; 63 University of Helsinki, Department of Geosciences and Geography, Helsinki, 00014 Finland; 64 The Natural History Museum, London, SW7 5BD UK; 65 Stockholm University, Department of Physical Geography, Stockholm, SE-106 91 Sweden; 66 Concordia University, Geography, Planning and Environment, Montreal, H3G 1M8 Canada; 67 Ca’ Foscari University of Venice, Department of Environmental Sciences, Informatics and Statistics, Venezia, 30172 Italy; 68 Herzen State Pedagogical University of Russia, Research Laboratory of the Environmental management, St. Petersburg, 191186 Russia; 69 Universitat de Barcelona, Departament de Biologia Evolutiva, Ecologia i Ciències Ambientals, Secció Ecologia, Barcelona, 08028 Spain; 70 University College Cork, Department of Geography, Cork, Ireland; 71 NORCE Norwegian Research Centre, LFI, Bergen, 5008 Norway; 72 US National Oceanic and Atmospheric Administration, National Centers for Environmental Information, Boulder, CO 80305 USA; 73 University of British Columbia, Department of Biology; Department of Earth, Environmental and Geographic Sciences, Kelowna, British Columbia V1V 1V7 Canada; 74 Landcare Research, Ecosystems and Conservation, Lincoln, 7640 New Zealand; 75 Chinese Academy of Sciences, Nanjing Institute of Geography and Limnology, Nanjing, 210008 China; 76 Institute of Archaeology and Ethnography, Russian Academy of Sciences, Siberian Branch, Novosibirsk 630090 Russia

Correction to: *Scientific Data* 10.1038/s41597-020-0445-3, published online 14 April 2020

In the original version of this Data Descriptor, the link to the metadata files produced during the journal’s curation process was incorrect. The correct link is 10.6084/m9.figshare.11967924

This has now been updated in the HTML version of the manuscript.

